# Safety and clinical efficacy of endoscopic procedures for the treatment of adjacent segmental disease after lumbar fusion: A systematic review and meta-analysis

**DOI:** 10.1371/journal.pone.0280135

**Published:** 2023-02-06

**Authors:** Nan Wang, Yimin Xie, Xiyu Liu, Yawei Zheng, Zhipeng Xi, Wenqiang Xu, Rongrong Deng, Tian Tang, Xin Liu

**Affiliations:** 1 Department of Spine Surgery, Affiliated Hospital of Integrated Traditional Chinese and Western Medicine for Nanjing University of Chinese Medicine, Nanjing, Jiangsu Province, P.R. China; 2 Department of Cardiovascular Medicine, Jiangsu Provincial Hospital of Traditional Chinese Medicine, Nanjing, Jiangsu Province, P.R. China; Stanford University School of Medicine, UNITED STATES

## Abstract

**Background:**

Adjacent segment disease (ASD) is a common complication after lumbar fusion and is still traditionally treated by open surgery. In recent years, with the development of minimally invasive techniques, percutaneous endoscopic surgery(PES) has been used for the treatment of ASD after lumbar fusion due to its unique benefits. Nevertheless, it remains unclear about its significant clinical efficacy and advantages over conventional open surgery.

**Objectives:**

To evaluate the clinical efficacy and safety of PES in the treatment of ASD after lumbar fusion.

**Study design:**

A systematic review and meta-analysis studies about the role of PES in managing ASD after lumbar fusion.

**Methods:**

A systematic search review was conducted in PubMed, EMBASE, Cochrane Library, Web of Science, CNKI, VIP, WanFang, and SinoMed databases from the start of their construction to 15 November 2021. Eligible studies included references to clinical trials of PES for ASD after open lumbar fusion. Observations included pain relief, recovery of postoperative function, overall excellent rates, and indicators of the advantages of minimally invasive surgery compared to conventional surgery. Postoperative complications and recurrence rates were also recorded.

**Results:**

A total of 24 studies, including 20 single-arm studies and 4 clinical control studies, all involving 928 patients were included. A total of 694 patients were included in the single-arm analysis. The results of the single-arm meta-analysis showed that PES could significantly reduce low back and leg pain and improve the functional status of the lumbar spine in patients with ASD after open lumbar fusion compared to preoperatively, and had good clinical efficacy after surgery. A total of 234 patients were included in the four clinically controlled studies, and the results of the meta-analysis showed that PES could clearly reduce pain and improve lumbar function, with no significant difference in efficacy between PES and open surgery. However, PES has a lower surgical incision, less intraoperative bleeding, and shorter operative time and length of hospital stay compared to open surgery. Moreover, it has a lower rate of postoperative recurrence as well as complications and a longer duration of efficacy.

**Conclusions:**

On the basis of the available clinical literature and the results of this study, PES could achieve satisfactory clinical effects in ASD treatment after lumbar fusion. Compared with conventional open surgery, PES can not only obtain similar clinical results, but also had the advantages of less trauma and faster recovery. Nevertheless, a randomized controlled study is still needed to validate the findings of this study.

**Trial registration:**

**Systematic review registration:** https://www.crd.york.ac.uk/prospero/, identifier CRD42022298387.

## Introduction

Currently, lumbar fusion is one of the most common procedures used to treat degenerative lumbar spine disease [1, 2]. The objective is to stabilize the diseased lumbar spine by surgical intervention. For a long time, fusion was considered the gold standard following decompression surgery. In the USA, the annual incidence of lumbar fusion procedures rose by 262% from 1998 to 2015 [3]. Moreover, with the growing amount of lumbar fusion being performed, the related medical costs have soared from US$3.7 billion in 2004 to US$10.2 billion in 2015, an impressive 177% increase, resulting in a major socioeconomic burden [4]. Despite this, lumbar fusion is associated with risk of complications [5], including degeneration of the adjacent segment [6].

Adjacent segment disease (ASD) is a broad concept that refers to a variety of complications in adjacent segments following spinal fusion, including disc disease, compression fractures, slippage, instability, and stenosis [7]. In the case of lumbar decompression surgery, which is essential to stable the spine, the segment can be well stabilized by fusion; however, spinal fusion increases the forces on the adjacent segment and compromised its flexibility [8]. Therefore, disease developing in the adjacent segment after lumbar fusion is relatively common. The progression of adjacent vertebral disease can lead to clinical symptoms that directly affect the fusion outcome, thus necessitating some patients to undergo re-surgical treatment [9, 10]. In patients with ASD requiring surgical management, open revision surgery has been the main therapeutic modality in the past, including extended decompression, adjunctive internal fixation, and further lengthening of the fused segment [11]. Most patients are hesitant to undergo open surgery again because of the painful experience, and the substantial trauma associated with open surgery causes scarring and fibrosis of the muscle tissue, which can affect the outcome of secondary surgery [12, 13].

Because of the advantages of minimum trauma, fast recovery, and a wide variety of reasons, percutaneous endoscopic surgery (PES) are increasingly routinely employed in patients with degenerative lumbar spine disease [14–17]. Clinical trials of endoscopic procedures for the treatment of ASD after lumbar fusion are already underway due to its multifaceted features. This study aims to provide clinicians with an objective assessment of the clinical outcomes of endoscopic procedures for the treatment of patients with ASD using a meta-analysis approach.

## Material and methods

This study has been registered in PROSPERO, and the registration number is CRD42022298387.

### Literature search and data sources

This systematic review was based on the systematic evaluation methodology and report quality as described in the PRISMA Guidelines [18] (S1 Checklist). A thorough literature review was conducted in PubMed, EMBASE, Cochrane Library, Web of Science, CNKI, VIP, Wanfang, and SinoMed databases related to endoscopic techniques for the treatment of ASD after lumbar fusion. Articles published before 15 November 2021 were referred to.

The search strategy, using Pubmed as an example, is as follows:

((adjacent level degeneration[All Fields] OR adjacent level disease[All Fields] OR adjacent level pathology[All Fields] OR adjacent segment degeneration[All Fields] OR adjacent segment disease[All Fields] OR adjacent segment pathology[All Fields]) OR (lumbar fusion[All Fields] OR ((Lumbar Vertebrae[MeSH] OR Lumbar Vertebrae[All Fields] OR Lumbar[All Fields]) AND (Spinal Fusions[MeSH] OR Spinal Fusions[All Fields] OR Fusion, Spinal[All Fields] OR Fusions, Spinal[All Fields])))) AND (Endoscopy[MeSH] OR Endoscopic surgery[All Fields] OR Surgical Procedures, Endoscopic[All Fields] OR Procedure, Endoscopic Surgical[All Fields] OR Procedures, Endoscopic Surgical[All Fields] OR Surgical Procedure, Endoscopic[All Fields] OR Endoscopy, Surgical[All Fields] OR Surgical Endoscopy[All Fields] OR Endoscopic Surgical Procedure[All Fields] OR Endoscopic Surgical Procedures[All Fields]).

### Study selection

To ensure the precision of this study, two reviewers independently screened the title and summary. The results of the search were imported into EndNote X9 software for further verification. The following criteria for studies were considered for their inclusion in the meta-analysis: (1) clinical research; (2) patients with ASD after lumbar fusion and an indication for surgery were included; (3) treatment of ASD using endoscopic techniques; (4) they reported at least one observation index among VAS (visual analog scale) scores (back or leg), JOA (Japanese Orthopedic Association) scores, ODI (Oswestry Disability Index), or MacNab efficacy evaluation criteria. Exclusion criteria included the following: (1) treatment process combined with other surgical procedures; (2) incomplete information or missing data; (3) duplicate publications, case reports and simultaneous submissions.

### Data extraction and quality assessment

Two authors worked independently to assess the quality of the included literature, extracting basic information and data for further analysis. If disagreements arose in the process, they were dealt with jointly after mutual consultation. Basic information extracted included the following: name of the first author, publication year, country, sample size, age and sex of patients, study design, duration of follow-up, surgical procedure, outcome indicators, postoperative complications, and recurrence rates. The quality of the included literature was assessed based on NOS (Newcastle-Ottawa Scale) tool [19].

### Data analysis

Stata 15 software was used for quantitative merging, where WMD values and 95% CI were set as the combined effect indicators, and the χ test combined with the I^2^ indicator was used to test the heterogeneity of the included studies. Studies were considered heterogeneous when P<0.1 and I^2^>50%, and the random-effects model was used. In contrast, no significant heterogeneity existed between studies when P≥0.1 and I^2^≤50%, and the fixed-effect model was used. Review Manager 5.4 was used as a heterogeneity test. Sensitivity analysis and subgroup analysis were performed to find the possible sources of heterogeneity. Publication bias was assessed by observing the symmetry of the funnel plot and Egger’s graph. In addition, meta-regression was used to analysis whether different observation nodes were a source of heterogeneity.

## Results

### Included studies

**Fig 1:**
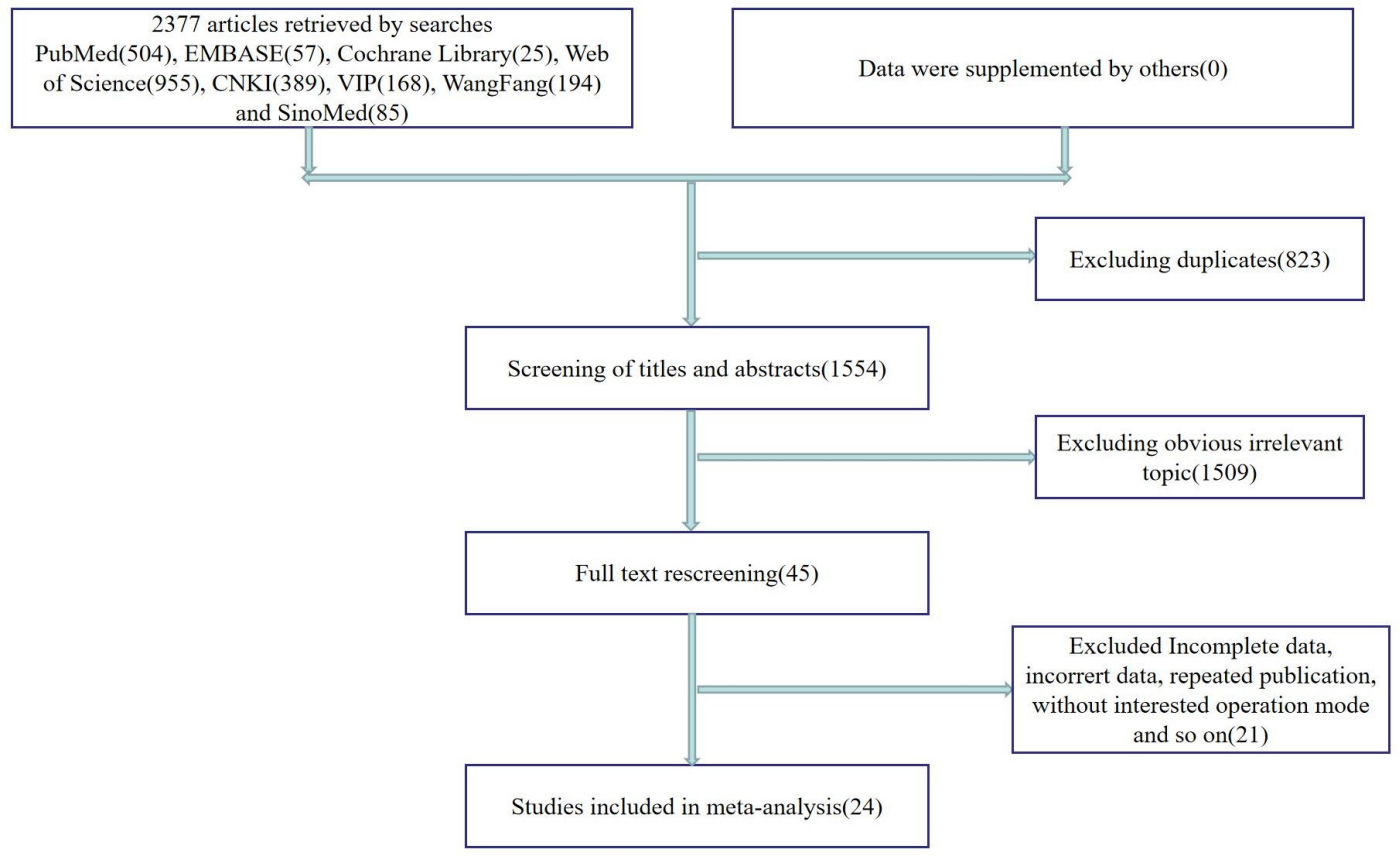
The specific screening process of this study.

Eight databases were searched according to the search strategy described in the Methods section, and 1954 studies were retrieved after de-duplication. Finally, a total of 24 clinical studies [15, 20–42] qualified for the meta-analysis. The specific screening process and results are shown in **Fig 1**. A total of 24 retrospective studies, including 20 single-arm studies and 4 case-control studies, were included in this study. Among the four control studies, all controls were open procedures, three had decompression fusion, and one had decompression only. The essential information for the included studies is summarized in **Table 1**, and the observation time nodes of different observation indices are summarized in **S1 Table in S1 File**. Of all the included studies, 20 were from China, 2 from Greece, 1 from the USA, and 1 from Japan.

**Table 1:** Basic characteristics of the 24 studies.

NO	Trial	Country	Study design	Sex(M/F)		Age		Treatment		Outcome	Follow-up (months)	NOS scores
				T	C	T	C	T	C			
1	Hiroki Iwai 2020 [20]	Japan	Retrospective study	10/3		64.8(45–83)		FESS		NRS, mJOA, SF-36	13.1	6
2	Xiaoming Liu 2019 [21]	China	Retrospective study	15/18		71(65–84)		FE-TF		VAS, mJOA, ODI	12–24	6
3	Guangfei Gu 2018 [22]	China	Retrospective study	11/14		74.65±9.61		PTED		VAS(back,leg), JOA, ODI, SF-36, MacMab	37.14±11.60	6
4	Albert Edward Telfeian 2017 [23]	USA	Prospective study	4/5		66.2(46–76)		PTED		VAS(back,leg), ODI	12	7
5	Wang Xuepeng 2019 [24]	China	Retrospective study	18/10		58.7±13.1		PTED		VAS(back,leg), ODI, MacMab	13.5	6
6	Liu Zuwang 2019 [25]	China	Retrospective study	161/42		63. 41±8.56		PTED		VAS, JOA, ODI, MacMab	12	5
7	Ma Jiye 2017 [26]	China	Retrospective study	24/18		45.39±2.07		PTED		VAS, JOA	6	6
8	Li Jianjiang 2015 [27]	China	Retrospective study	23/8		66.2(45–81)		PTED		VAS, JOA	6	6
9	Zheng Zhenyang 2016 [28]	China	Retrospective study	9/13		54.9(42–63)		PTED		VAS(back,leg), ODI, MacMab	14.4	6
10	Wang Xiaoli 2021 [29]	China	Retrospective study	11/8		54.8±11.23		PELD		VAS(back,leg), ODI, MacMab	22.56±6.72	6
11	Xu Feng 2018 [30]	China	Retrospective study	13/18		65.5(43–85)		PELD		VAS, JOA, MacMab	18.3	6
12	Fu Song 2019 [31]	China	Retrospective study	29/18		64.91±7.12		PTED		VAS(back,leg), ODI, MacMab	14.22±2.34	6
13	Wang Zhen 2018 [32]	China	Retrospective study	31/19		63.4±12.6		PTED		VAS(back,leg), JOA, ODI	6	6
14	Huang Shenchang 2017 [33]	China	Retrospective study	12/16		62.9±7.5		PTED		VAS(back,leg), ODI, MacMab	25.1±3.4	6
15	Zhang Jianjun 2020 [34]	China	Retrospective study	9/6		68(64–75)		PTED		VAS, JOA, ODI, MacMab	12.5±7.2	6
16	Liu Pengfei 2013 [35]	China	Retrospective study	8/12		52.5(45–60)		PTED		VAS(back,leg), MacMab	18.3(12–23)	6
17	Gao Kun 2018 [36]	China	Retrospective study	12/8		63(58–68)		PTED		VAS, JOA, ODI	6–18	6
18	Zhong Honghua 2020 [37]	China	Retrospective study	21/15		59.45±4.59		PTED		VAS, ODI	-	6
19	Zhaoyu Ba 2017 [15]	China	Retrospective study	18/15	10/21	70.8(65–81)	68.7(64–78)	PETF	PLF	JOA, ODI	12–24	9
20	Tong Li 2020 [38]	China	Retrospective study	18/12	19/15	61.0±14.3	62.9±10.7	FELD	PLF	VAS(back,leg), JOA, ODI	32.1±6.0	9
21	Fu Zhongquan 2019 [39]	China	Retrospective study	16/15	16/16	45.5±4.56	49.6±5.03	PTED	PLF	VAS(back,leg), ODI	12.7±2.21	9
22	Li Peng 2021 [40]	China	Retrospective study	18/8	11/6	53.88±13.69	54.17±15.03	PTED	SP	VAS, ODI	6–12	8
23	Stylianos Kapetanakis 2020 [41]	Greece	Prospective study	8/7		57.2±2.2		PTED		VAS(back,leg), SF-36	-	8
24	Stylianos Kapetanakis 2021 [42]	Greece	Prospective study	3/4		59.6		PTED		VAS, ODI	16.8	7

Note: M: male; F: female; T: treatment; C: control; NOS: Newcastle-Ottawa Scale; FESS: full-endoscopic spine surgery; FETF: Full-endoscopic transforaminal procedure; PTED: Percutaneous Transforaminal Endoscopic Discectomy; PELD: Percutaneous Endoscopic Lumbar Discectomy; PLF: posterior lumbar fusion; SP: Simple open surgery; NRS: Numerical Rating Scale; mJOA: modified Japanese Orthopaedic Association; VAS: Visual analog scale; ODI: Oswestry disability index; JOA: Japanese Orthopaedic Association.

### Study quality

The results of the NOS tool for literature quality assessment are shown in **Table 1**. Among the 24 studies selected, 21 were retrospective studies. The NOS score of 3 studies was 9, 1 study was 8, 16 studies was 6, and 1 study was 5. Out of 3 prospective studies, 1 study had a score of 8, and 2 studies had a score of 7.

### Meta-analysis of postoperative outcomes

After careful reading and analysis of the 24 included publications, we summarized the evaluation indicators for the efficacy and safety of PES for the treatment of ASD after lumbar fusion. The VAS score was a pain-related indicator, the ODI and JOA scores were for lumbar spine function, and the MacNab criterion was for overall outcomes. Furthermore, the minimally invasive features of PES were the surgical incision, the amount of intraoperative bleeding, the duration of surgery, and the length of hospital stay. In addition, we also counted the number of patients with complications and the number of patients showing ASD recurrence after PES. A major postoperative evaluation indicator is the VAS score, which is considered the gold standard for quantifying pain.

### Pain relief

VAS score is an important indicator of postoperative pain relief. For PES study, 12 studies involving 324 patients explicitly divided the VAS score into VAS-back and VAS-leg scores. A single-arm meta-analysis showed a significant reduction in postoperative pain scores compared to the preoperative period, both in the VAS-back score (WMD: -3.87; 95% CI: [-4.33, -3.41], I^2^ = 97.1%), and the VAS-leg score (WMD: -5.06; 95% CI: [-5.49, -4.64], I^2^ = 96.5%). Ten studies involving 444 patients mentioned only VAS scores without specific sites. A single-arm meta-analysis showed a significant reduction in VAS scores (WMD: -5.59; 95% CI: [-5.94, -5.23], I^2^ = 97.6%) compared to the preoperative period. There were obvious heterogeneity among this studies (I^2^>50%), so random effect models were selected for analysis.

**Fig 2:**
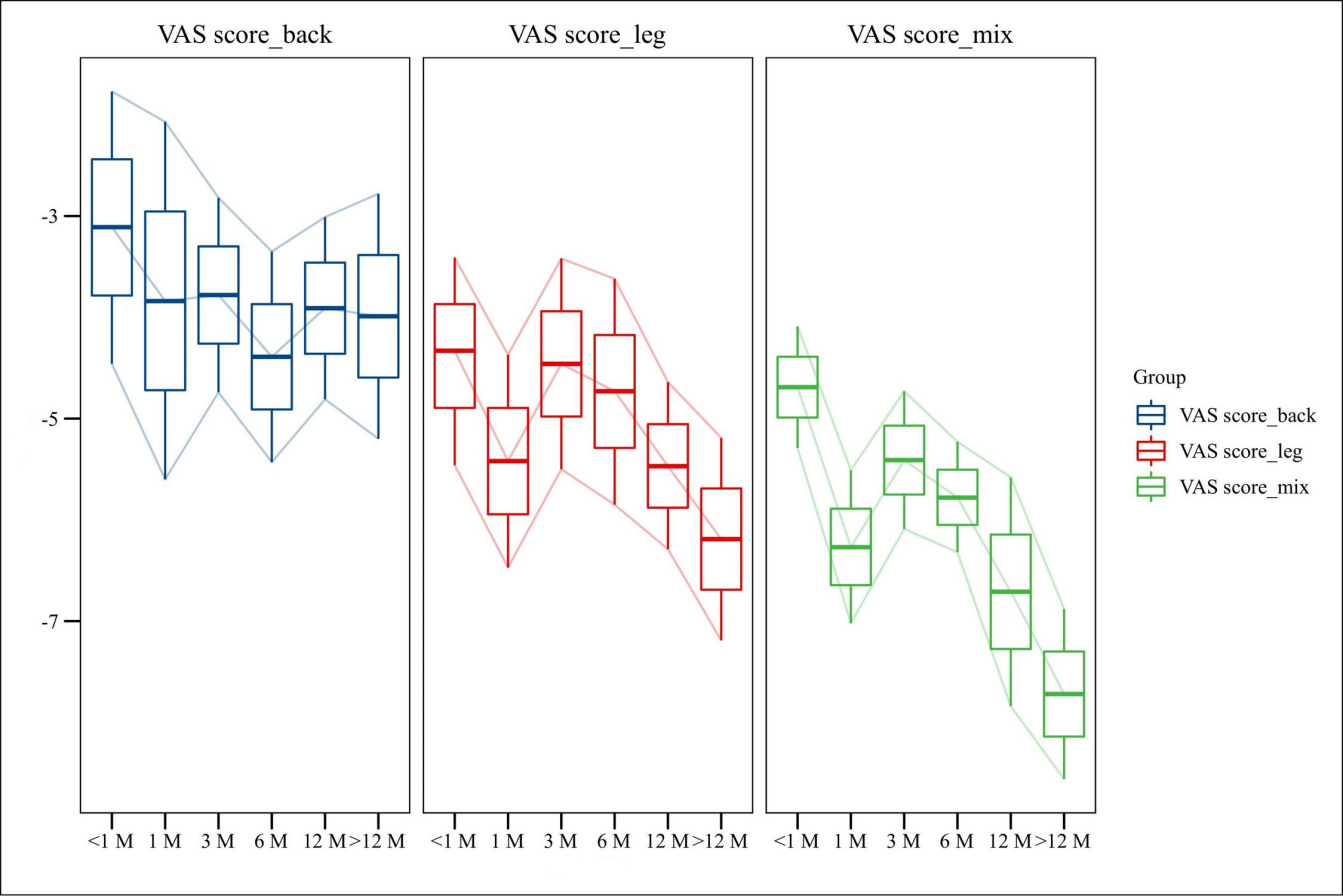
Development trend of postoperative VAS score.

In this study, the observation nodes of postoperative pain in patients with ASD after lumbar fusion treated with PES were divided into 6 parts: less than 1 month, 1 month, 3 months, 6 months, 12 months, and finally the follow-up time (>12 months) after surgery. The results showed a gradual trend towards further relief of both postoperative low back pain and leg pain as the follow-up period increased (**Fig 2**).

**Fig 3:**
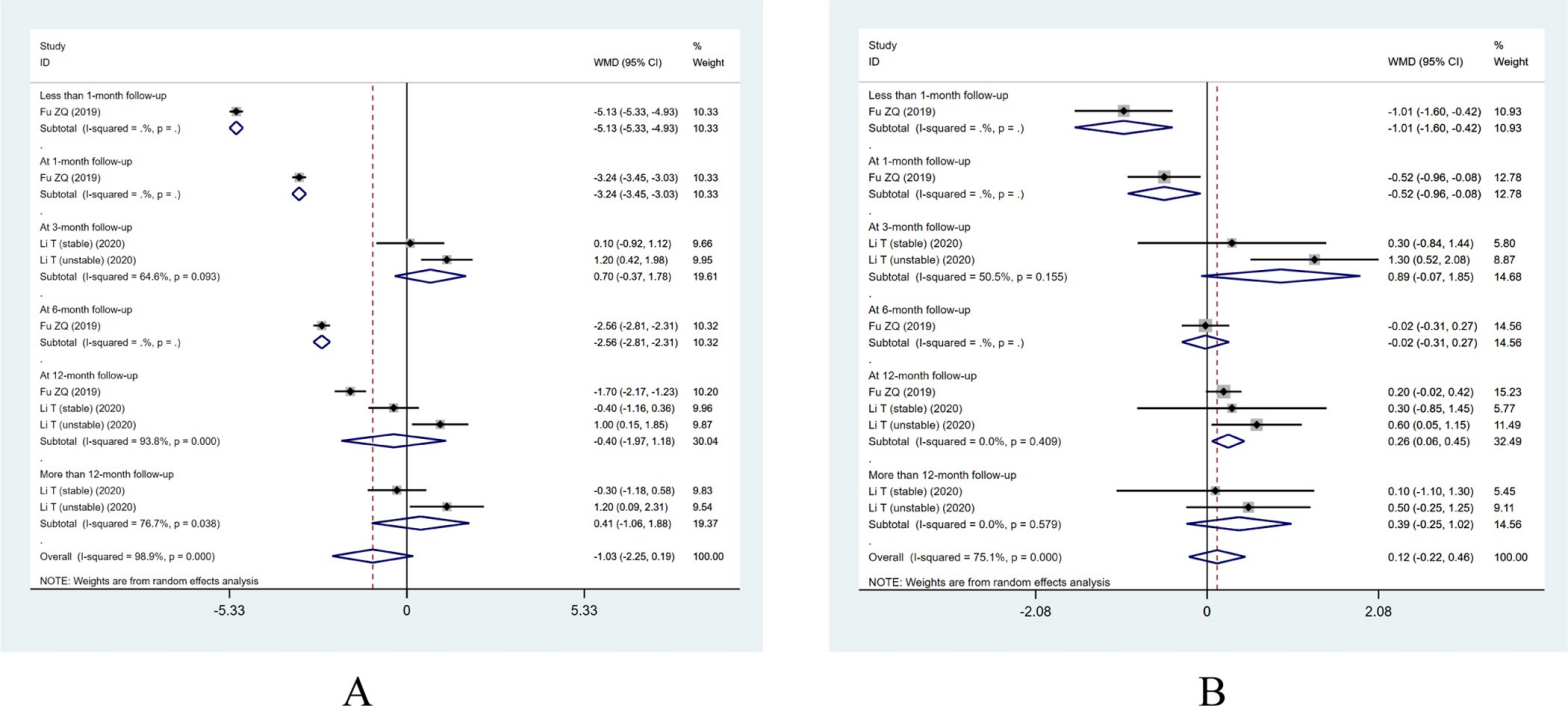
Forest plot of VAS scores. (A) Forest plot of clinical controlled trials of VAS-back scores; (B) Forest plot of clinical controlled trials of VAS-leg scores.

A total of four case-control studies were performed, including one case in which the VAS score was mentioned without specifying the site and two cases in which the VAS-back and VAS-leg scores were divided. Meta-analysis revealed a non-significant difference in terms of the VAS-back score (WMD: -1.03; 95% CI: [-2.25, 0.19], I^2^ = 98.9%) (**Fig 3A**) and the VAS-leg score (WMD: 0.12; 95% CI: [-0.22, 0.46], I^2^ = 75.1%) in the PES group compared to the open surgery group (**Fig 3B**). There were obvious heterogeneity among this studies (I^2^>50%), so random effect models were selected for analysis.

### Functional recovery

#### JOA score

**Fig 4:**
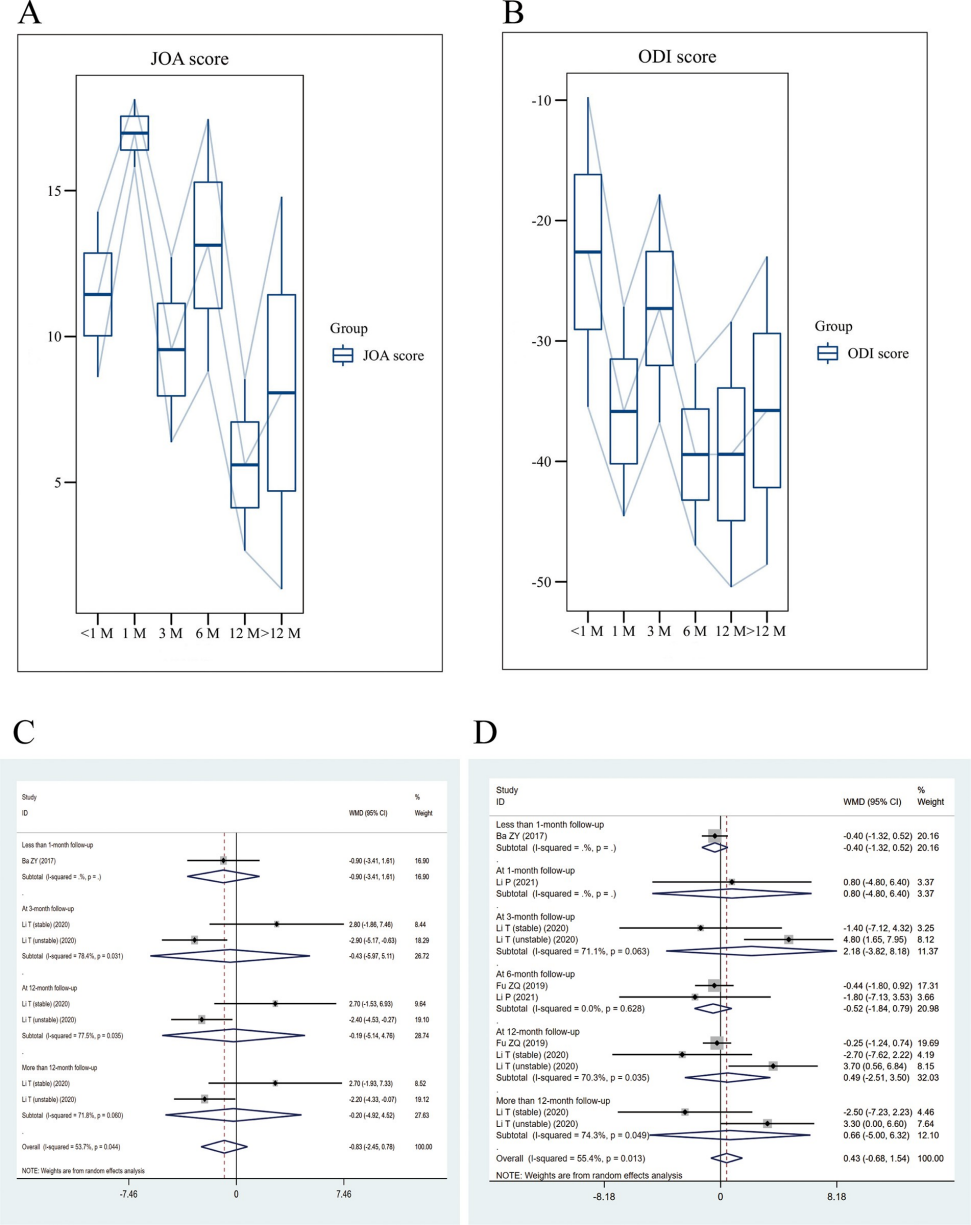
Trend plots for single arm meta-analysis of JOA and ODI and clinical control forest plots. **(A)** Postoperative JOA trend chart; **(B)** Postoperative JOA forest plot about clinical trials; **(C)** Postoperative ODI trend chart; **(D)** Postoperative ODI forest plot about clinical trials.

Ten of the included studies contained JOA scores. The results of the single-arm meta-analysis showed that PES significantly improved JOA scores in postoperative patients (WMD: 10.67; 95% CI: [8.85, 12.50], I^2^ = 97.9%) (**Fig 4A**). There was obvious heterogeneity in this study (I^2^>50%), so random effect model was selected for analysis. Two studies conducting case-control trials and meta-analyses showed that both PTED and open surgery improved patients’ postoperative JOA scores (WMD: -0.83; 95% CI: [-2.45, 0.78], I^2^ = 53.7%) (**Fig 4B**); however, no significant difference was observed between the two comparisons.

#### ODI score

Seventeen of the included studies contained ODI. The results of the single-arm meta-analysis showed that PES significantly reduced ODI scores in postoperative patients (WMD: -33.38; 95% CI: [-37.19, -29.54], I^2^ = 99.4%) (**Fig 4C**). There was obvious heterogeneity in this study (I^2^>50%), so random effect model was selected for analysis. Moreover, no significant differences were observed in ODI scores (WMD: 0.43; 95% CI: [-0.68, 1.54], I^2^ = 55.4%) (**Fig 4D**) between PTED and controls in the four case-control trials.

#### Overall outcome

**Fig 5:**
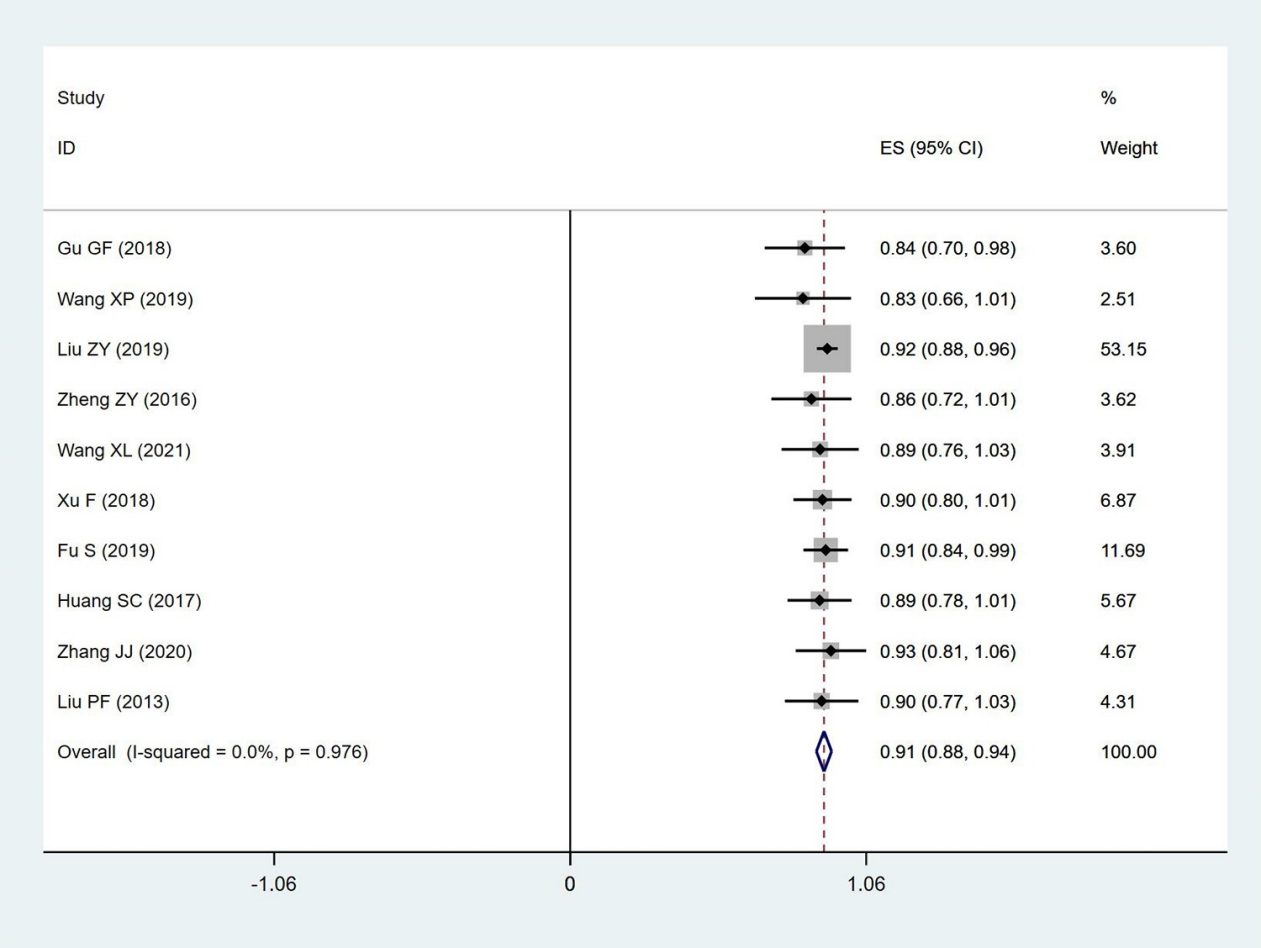
Forest plot of MacNab criterion.

The MacNab criterion, an indicator of the overall outcome of postoperative patients, was mentioned in 10 studies. The results of the meta-analysis indicated that PES for ASD after lumbar fusion could achieve a high rate of improvement (ES: 0.91; 95% CI: [0.88, 0.94], I^2^ = 0.0%). The MacNab criterion attained an excellent rate of over 90% (**Fig 5**).

#### Meta-analysis of surgical indicators

**Fig 6:**
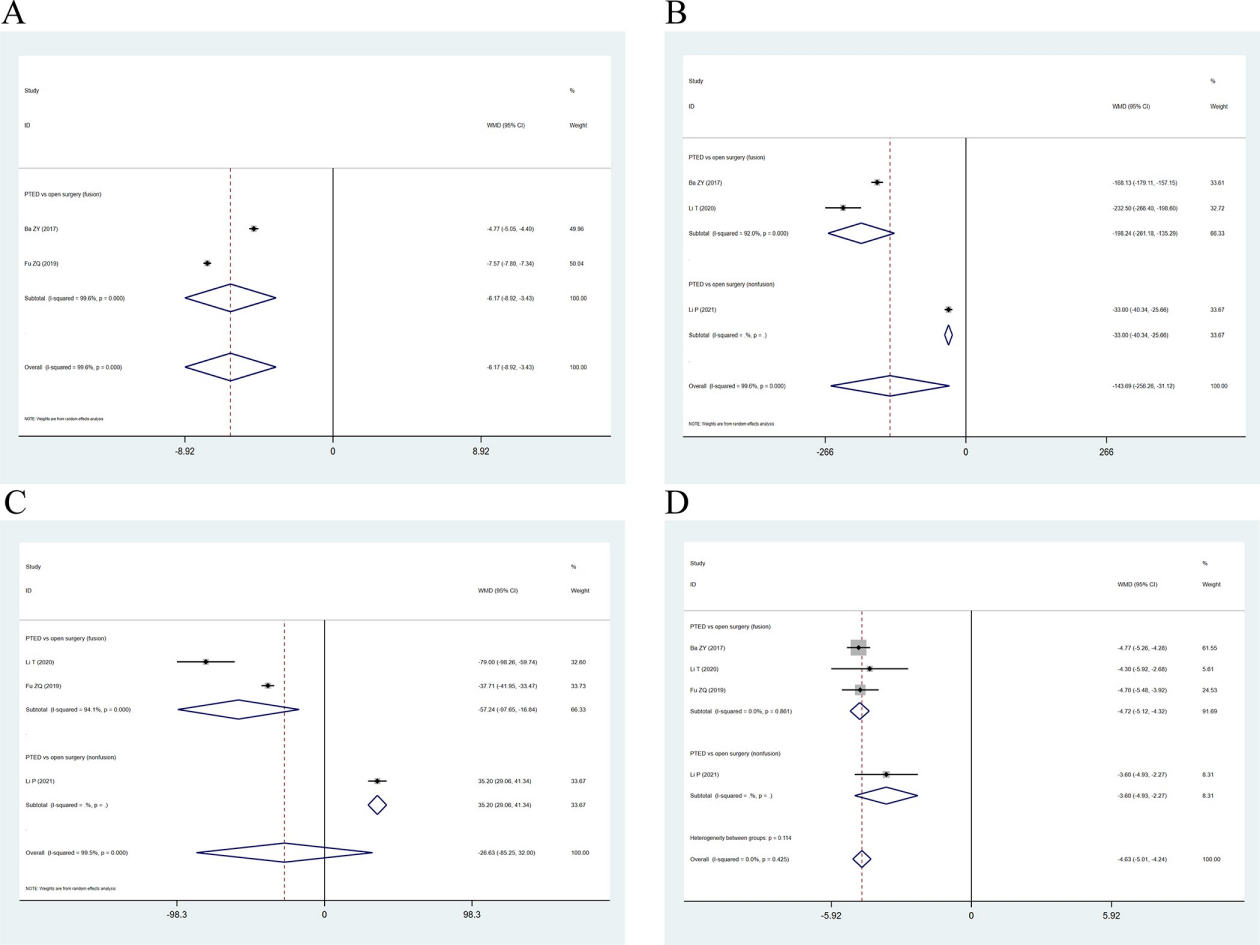
Forest plots for surgical indicators. **(A)** The length of skin incision; **(B)** The intraoperative bleeding; **(C)** The operative time; **(D)** The hospitalization time.

Two studies referred to the length of the skin incision. Meta-analysis results showed that PTED has a smaller incision (WMD: -6.17; 95% CI: [-8.92, -3.43], I^2^ = 99.6%) (**Fig 6A**) in the treatment of ASD compared to open surgery. There was obvious heterogeneity in this study (I^2^>50%), so random effect model was selected for analysis. Three studies referred to the blood loss. The results of the meta-analysis showed that PTED has less bleeding (WMD: -143.69; 95% CI: [-256.26, -31.12], I^2^ = 99.6%) (**Fig 6B**) compared to open surgery in the treatment of ASD after lumbar fusion. There was obvious heterogeneity in this study (I^2^>50%), so random effect model was selected for analysis. Three studies referred to the operative time. The results of the meta-analysis showed that there was no significant difference between PTED and open surgery in terms of operative time (WMD: -26.63; 95% CI: [-85.25, 32.00], I^2^ = 99.5%) (**Fig 6C**), the random effect model was used to analyze the data. This may be attributed to the different ways in which open surgery is performed. Excluding non-fusion surgery, two studies reported a comparison between fusion surgery and PTED. The results of the meta-analysis indicated that PTED surgery required a shorter operative time (WMD: -57.24; 95% CI: [-97.65, -16.84], I^2^ = 94.1%) (**Fig 6C**) than fusion surgery. Because of the high heterogeneity of these surgical indicators, we selected random effect model to analyze the data. The source of these heterogeneity can be related to different surgical methods, operators and others. Four studies reported on hospitalization time. The results of the meta-analysis showed that PTED has a shorter hospitalization time (WMD: -4.63; 95% CI: [-5.01, -4.24], I^2^ = 0.0%) (**Fig 6D**) compared to open surgery in the treatment of ASD.

#### Complications

**Fig 7:**
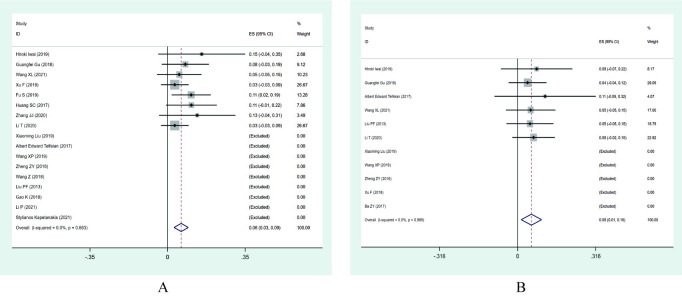
Forest plot of complications and recurrence rates after PES. (A) Complications; (B) Rescurrence rates.

Seventeen studies documented the presence or absence of postoperative complications, out of which nine reported no significant complications. Meta-analysis results indicated that the incidence of postoperative complications after PES for ASD was approximately 6% (ES: 0.06; 95% CI: [0.03, 0.09], I^2^ = 0.0%) (**Fig 7A**). A total of 423 patients were treated with PES in these 17 studies, with 17 patients reporting the occurrence of postoperative complications. The main postoperative complications were dural sac injury, cerebrospinal fluid leakage, infection, and nerve damage, of which two patients were accurately mentioned as having cerebrospinal fluid leakage.

#### Recurrence

Eleven studies reported postoperative recurrence, five of which reported no significant recurrence. Meta-analysis indicated a low recurrence rate of roughly 6% (ES: 0.06; 95% CI: [0.01, 0.10], I^2^ = 0.0%) (**Fig 7B**) after PES for ASD. A total of 263 patients were treated with PES in these 11 studies, and 7 patients with postoperative recurrence were reported.

#### Publication bias

**Fig 8:**
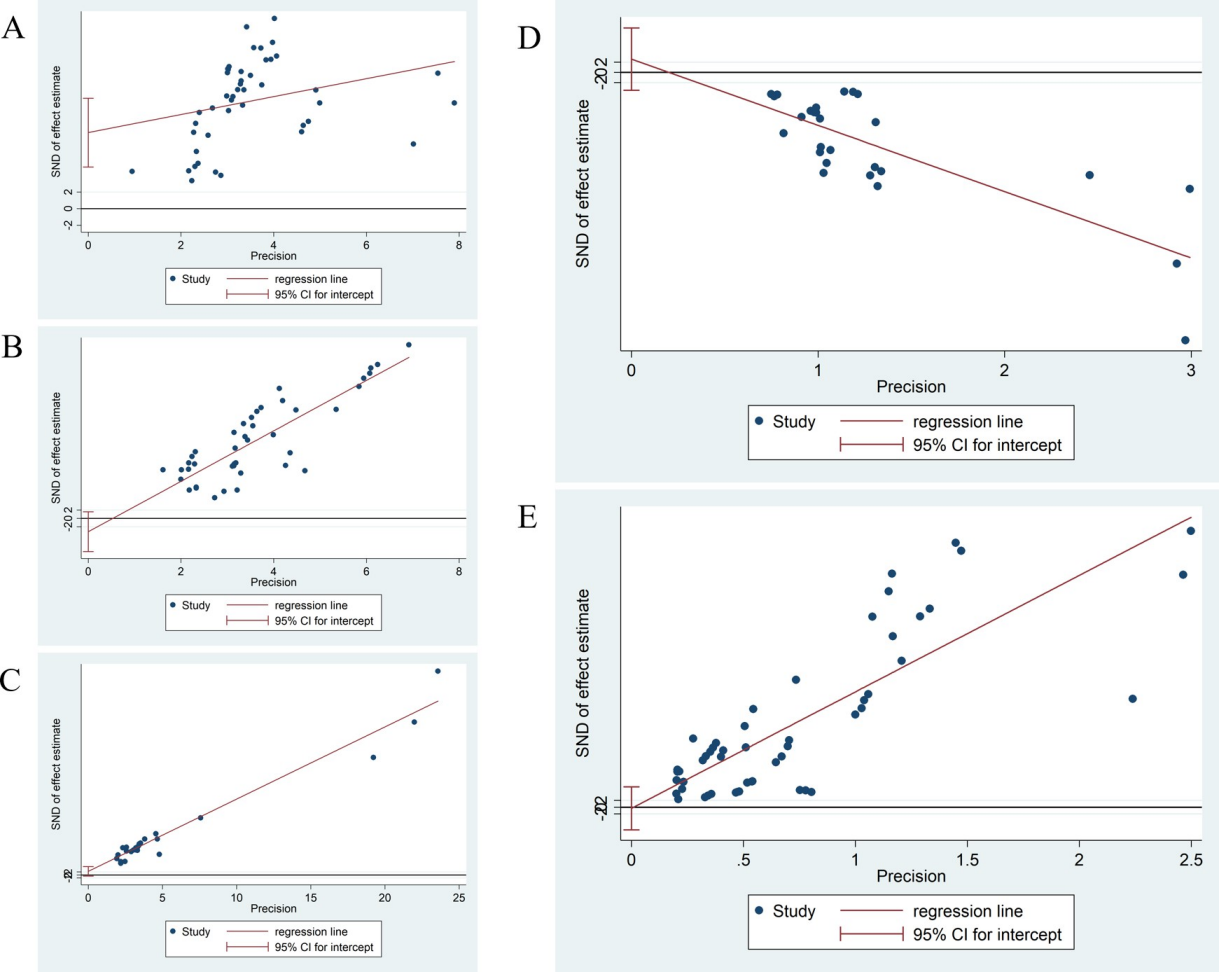
Egger graph of postoperative outcomes after PES. **(A)** Egger graph of VAS-back; **(B)** Egger graph of VAS-leg; **(C)** Egger graph of VAS-mix; **(D)** Egger graph of JOA; **(E)** Egger graph of ODI.

The funnel plot allows for some evaluation of the publication bias of the study. Its bias can be evaluated by visual observation of the position of the points on the funnel plot. Funnel plots were conducted for different indicators of observation, and all were found to have some given bias, which may be connected to the small sample size, the low quality of the literature, and the different methods of evaluation by different researchers (**S1 Fig**). Due to the asymmetry of funnel graph, we also conducted Egger’s test on several main indicators to evaluate publication bias. The results showed that the VAS-back value (**Fig 8A**) (P = 0.000<0.05) was asymmetric at the regression line; VAS-leg value (**Fig 8B**) (P = 0.180>0.05), VAS-mix value (**Fig 8C**) (P = 0.139>0.05), JOA value (**Fig 8D**) (P = 0.383>0.05) and ODI value (**Fig 8E**) (P = 0.910>0.05) were almost symmetrical on both sides of the regression line, indicating that the possibility of publication bias was low.

#### Sensitivity analysis

To further explain the high heterogeneity of the results, we performed a sensitivity analysis. However, we did not find any positive changes in the results using this method (**S2 Fig**). In addition, we also tested for heterogeneity in several key indicators through Revman 5.4, looking for literature that might have a significant impact on the heterogeneous results by removing one of the studies individually. After analysis we did not find a referenceable result (**S3 Fig**).

#### Meta-regression analysis

A meta-regression analysis was done using the time observation node in the study to clarify whether it influenced the results of this study. Meta-regression analysis for VAS-back, JOA and ODI showed that the p-value for the time nodes were greater than 0.05, indicating that they did not significantly influence the results of VAS-back. The meta-regression analysis of the VAS-leg and VAS-mix showed a p-value of less than 0.05 for the time node, indicating that it had some effect on the results of these two observations (**S2 Table in S1 File**).

## Discussion

ASD, a common complication following lumbar spine fusion, is primarily defined as a painful disorder of the low back and legs caused by fusion of a segment with degeneration of the adjacent segment [43]. The onset of ASD after lumbar fusion is generally treated conservatively for symptomatic relief, but when symptomatic treatment is inadequate or obvious surgical indications exist, lumbar interbody fusion is a popular type of surgery. However, patients who have undergone lumbar fusion are usually reluctant to undergo it again. In particular, the scarring of the tissue from the previous fusion surgery makes reoperation difficult, and the damage to the vertebral motion can lead to further ASD and other complications [44]. In contrast, PES is used in the treatment of ASD after lumbar fusion because of its advantages of less trauma, faster recovery, and fewer complications. Moreover, its effectiveness and safety have been demonstrated in previous clinical studies [15, 20–42]. Nonetheless, studies on PES for ASD remain scarce, and no systematic review to prove its effectiveness exists. To our knowledge, this study is the first to prove the effectiveness and safety of PES using meta-analysis and systematic evaluation, providing a higher level of evidence.

This study comprised a total of 24 studies, 20 single-arm studies, and 4 clinical control studies, involving a total of 928 patients. The PES included here were all fully endoscopic decompression-only procedures, of which the PTED procedure was the predominant one. Of the open procedures selected for the control group, three studies used posterior lumbar fusion (PIF) and one study used simple open subtotal decompression surgery. Eight-hundred and fourteen patients were treated with minimally invasive surgery, and one-hundred and fourteen patients were treated with open surgery. Ninety-seven cases of decompression fusion and seventeen cases of simple decompression were included in the open surgery. This single-arm meta-analysis showed that PES significantly reduced preoperative low back and leg pain and improved lumbar spine function in patients, resulting in good excellent rates. Additionally, meta-analysis results showed that PES could achieve comparable clinical outcomes compared to open surgery. Moreover, it resulted in smaller incisions, lesser bleeding, shorter operation times and hospital stays compared to open surgery. In the included literature, we also counted the complications and recurrences of PES for ASD, and the results showed a low number of complications and a minimal recurrence rate. It is evident that PES is clinically effective and safe in the treatment of ASD; in addition, PES is more minimally invasive than traditional open surgery.

Due to the high heterogeneity, a subgroup analysis of patient follow-up times was performed, and patients were mainly divided into postoperative, 1-month postoperative, 3-month postoperative, 6-month postoperative, 12-month postoperative, and final follow-up (>12 months). A high level of heterogeneity was found in the results, showing that the duration of follow-up was not the main source of heterogeneity. The results of the subgroup analysis showed a consistent trend of decreasing VAS scores (no matter than back or leg) and ODI scores in the single-arm study patients as the time after surgery increased. The JOA scores showed a consistent upward trend until December, but decreased in December and beyond, which may be related to the inclusion of less literature, higher heterogeneity, and other factors. Notably, PES can maintain its long-term efficacy in the treatment of ASD. Since 3 of the 4 included control studies were open plus fusion surgery and one was open surgery alone, this remarkably affected the observed indicators of the procedure. Therefore, we performed a subgroup analysis. The results showed that the bleeding and length of stay for open surgery alone was more than for PES but less than for PLF, and that open surgery alone was the shortest in terms of operative time. However, due to the small volume of literature included, the credibility of this finding needs to be further validated in future studies.

A sensitivity analysis was carried out to find possible causes of the high heterogeneity. The results did not reveal articles that caused significantly higher heterogeneity. We speculated that the different follow-up times might be important in causing the elevated heterogeneity, so we further performed a meta-regression analysis of the time observation nodes and found no significant differences in any of the other indicators, although VAS-leg and VAS-mix were found to be possibly associated with it. We speculate a high level of heterogeneity may occur due to the operator’s level of surgery, different observation environments, research staff, and complex surgical situations. Hu Zhenxin et al. [45] also found in their meta-analysis that when looking at conditions such as low back pain and lumbar spine function, the VAS and ODI always showed high heterogeneity, which is a possible reason for the high heterogeneity in this article. Due to clinical and methodological factors, the meta-analysis can have a high level of heterogeneity in terms of observed indicators [46]. Therefore, in order to reduce heterogeneity, later clinical studies need to make some improvements, such as standardizing the follow-up time, focusing on the primary outcome, and standardizing the surgical approach.

With advances in minimally invasive surgery, PES has become the mainstream treatment for lumbar degenerative diseases such as lumbar disc herniation and lumbar spinal stenosis owing to its advantages of minimal invasiveness, a quick recovery, less payment, and few complications [47–49]. Although it is the strategy of choice for the treatment of LDH [50], it has been gradually used in the treatment of ASD in recent years. PES allows precise access to the lesion and achieves good decompression with less disruption to the overall structure of the spine [51]. Many patients with ASD experience pain after extended open fusion surgery and still require painkillers [52]. This single-arm meta-analysis demonstrates the clinical efficacy of PES for ASD in relieving pain and improving lumbar spine function. Moreover, the findings show that PES remains effective with longer follow-up. The results of the control study also showed that PES could achieve clinical results comparable to PLF, with no significant difference in the meta-analysis comparison. However, there is no doubt about its minimally invasive nature. In the treatment of degenerative lumbar spine disease, the currently accepted clinical goals are minimal trauma, optimal neurological decompression and maximum preservation of the lumbar segmental motion [53]. In this context, PES is gradually gaining acceptance among clinicians and expanding the scope of its clinical use. A multicentre randomised controlled study by Aichmair et al. [54] found that PES achieved ideal nerve root decompression, with intraoperative nerve root decompression resulting in a large local cushion space, which allowed the nerve roots to relax, preserving excellent lumbar mobility and achieving favorable clinical outcomes.

In addition, re-fusion surgery in patients with ASD is difficult and increases the risk of complications, such as further ASD, incomplete fusion, fusion displacement, nerve damage, retroperitoneal hematoma [13]. However, postoperative complications after PES are less common. The results of this study showed that the postoperative complication rate for PES is 4.02%, with the most frequent complication being a torn dural sac, which generally has a good prognosis. Evidently, PES not only achieves a good clinical outcome, but also has a low recurrence rate and fewer postoperative complications.

Previous studies have found that the postoperative recurrence rate for PES is approximately 0–6.9% [55]. The findings of this study also suggest that PES has a postoperative recurrence rate of approximately 2.66% in the treatment of ASD, which is not inconsistent with previous studies. Although previous studies have shown that PLF for ASD has a lower postoperative recurrence rate [56], the physical and psychological burden of lumbar fusion on the patient and the risk of postoperative complications should be taken into account. Therefore, PES may be a better option for ASD management.

The 10-year incidence rate of ASD is 0–27% after initial fusion surgery [57]. Furthermore, ASD is a normal degenerative process that is mostly visible on imaging markers; nevertheless, when patients become symptomatic, conservative therapy should be sought initially, and surgical options should be carefully examined. In this group of patients, fusion is currently the surgical procedure of choice, but most patients with the successful fusion of adjacent segments may continue to develop the disease in subsequent adjacent segments, further exacerbating the risk of ASD [58]. Even with the rapid development of minimally invasive technology, minimally invasive fusion techniques such as minimally invasive transforaminal lumbar interbody fusion (MIS-TLIF) [59, 60] and endoscopic lumbar interbody fusion (Endo-LIF) [61] have been widely used in clinical practice. They offer a variety of minimally invasive advantages, but the stability of the spinal segment after fusion will inevitably affect adjacent segments. Therefore, it is necessary to ensure the protection of the biomechanical environment of the spine while performing this technique.

In addition to the operator’s skills, the relevant indications are crucial to assuring the success of PES in the treatment of ASD. Patients with predominantly neurological compression symptoms can be relieved by endoscopic decompression, but the procedure is performed in a narrow channel with limited access to the lesion target and operating space and is contraindicated in cases of severe lumbar instability and severe intradural adhesions [62]. The procedure is not suitable for patients with congenital spinal instability and excessive loss of spinal space height, as excessive decompression may affect the stability of the lumbar spine. For procedures with extensive and difficult exposure, such as tumors, which cannot be solved by PES; the presence of a surgical history in the responsible segment and the skill of the surgeon can also affect the outcome of the procedure; the accuracy of the puncture point and the microscopic manipulation of the patient are also particularly crucial during the procedure. Patients with combined lumbar instability, lumbar spondylolisthesis, severe neurological damage and infection will still require open surgery for revision or extended fixation of the fused segment. Additionally, in the case of severe lumbar spinal stenosis with instability, slippage, or isthmic fracture of the diseased segment, the endoscopic fusion technique has shown significant minimally invasive advantages compared to other types of lumbar spine surgery, in addition to quick recovery, less hospital stay, and lower costs [63, 64].

However, PES is not without limitations. It requires a long learning curve, a clear knowledge of anatomy, and expertise. The therapeutic value of PES in ASD can only be achieved if the appropriate indications for the procedure are mastered and the surgeon is well trained.

## Limitation

Although this study is the first systematic evaluation and meta-analysis of minimally invasive surgery for ASD, it still has some limitations. First, none of the included literature was a randomized controlled study, making the results somewhat restricted. Second, the sample sizes included in most studies were excessively small, this makes the results obtained in this article subject to some error. Third, although four trials of controlled studies were included, the volume of articles was relatively small, and there was no fixed control surgical procedure, which still needs to be validated in clinical studies by designing large-scale multicentre randomized controlled trials. Fourthly, in terms of risk of bias analysis, this article was found to be at high risk; the quality of included studies was inadequate; and there was a high level of heterogeneity in the results, which are reasons why the results of the meta-analysis are not convincing. Fifth, due to clinical and methodological factors, the heterogeneity of the observations in this paper is high, and the main cause of the heterogeneity was not found by multiple methods, which needs to be further improved later. Ultimately, endoscopic decompression alone increases the risk of segmental instability, a condition that can be manifested even years after surgery by clinical symptoms and on radiographic imaging, so the follow-up period for the studies included here remains short, which can have an important impact on the results.

## Conclusion

This systematic review demonstrates that PES treatment for ASD can be clinically effective, with the advantages of being minimally invasive, having few complications, and having a low recurrence rate. The advantages of PES in the treatment of ASD are obvious, but the level of evidence in this study is not high and the literature presented is mostly retrospective studies and single-arm trials, with rigorous randomized controlled trials still to be conducted in the future to demonstrate its efficacy. The results in this article have a high degree of heterogeneity and are an area for improvement at a later stage.

## Supporting information

S1 Checklist PRISMA 2020 checklist. (DOCX)

S1 Fig Funnel chart analysis of multiple indicators. (PDF)

S2 Fig Sensitivity analysis of multiple indicators. (PDF)

S3 Fig Heterogeneity tests by forest plots. (PDF)

S1 File S1 Table. Observation time nodes of different observation indexes in the 24 studies. S2 Table. Meta-regression analysis of observation time nodes in this study.
(DOC)
