# Supplementary material for: Safety and clinical efficacy of endoscopic procedures for the treatment of adjacent segmental disease after lumbar fusion: A systematic review and meta-analysis
Source: PLoS One. 2023 Feb 6;18(2):e0280135. doi: 10.1371/journal.pone.0280135 (PMC9901788; doi:10.1371/journal.pone.0280135)
Supplement: S1 Fig — (PDF) [file pone.0280135.s002.pdf]

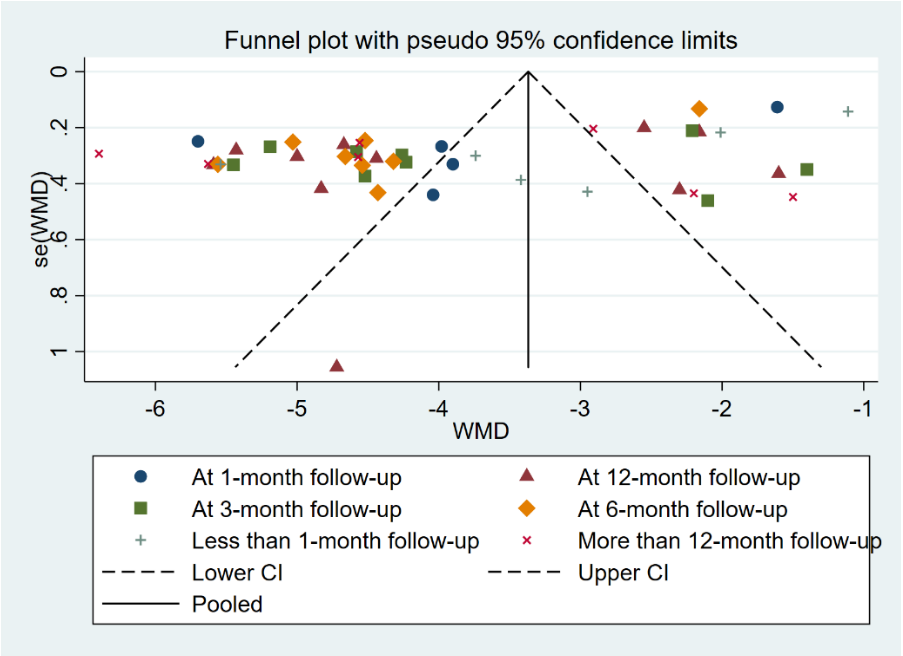

FIGURE 1 Vas-back grouping funnel plot

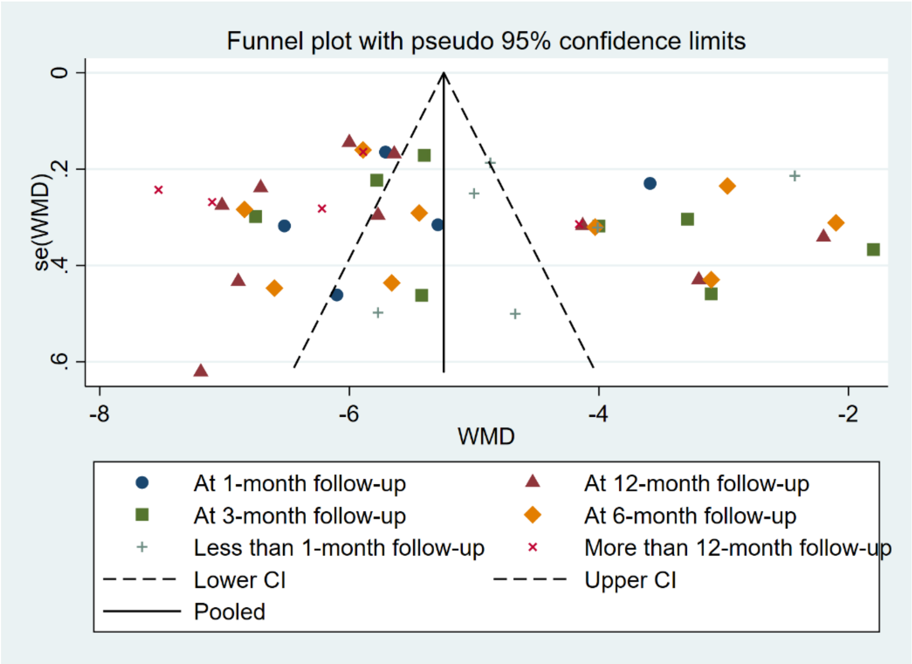

FIGURE 2 Vas-leg grouping funnel plot

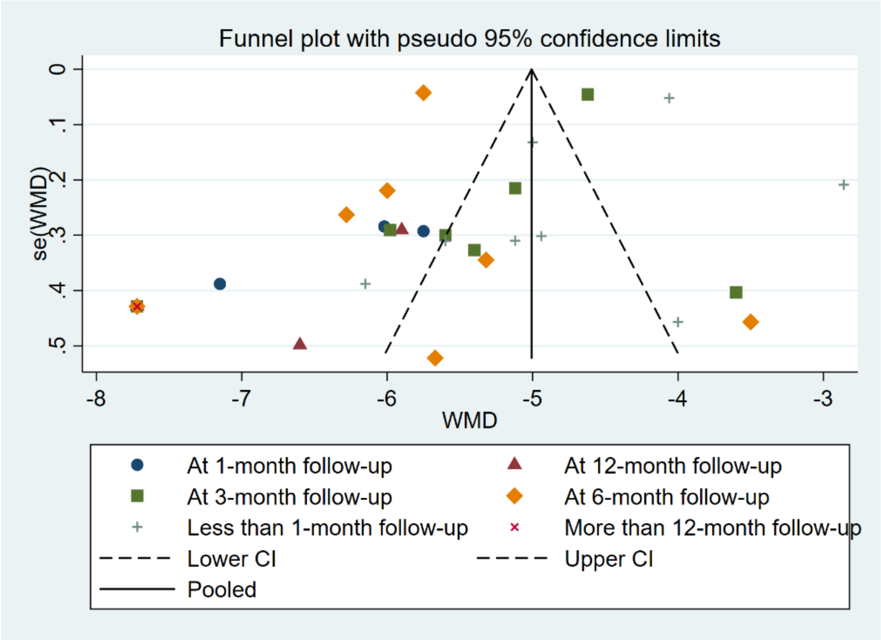

FIGURE 3 Vas-mix grouping funnel plot

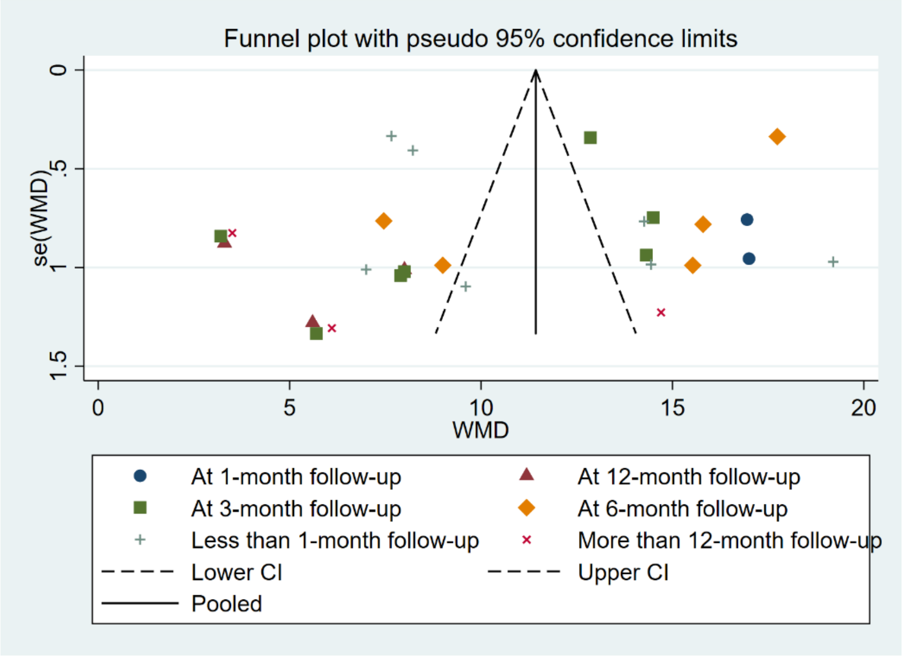

FIGURE 4 JOA grouping funnel plot

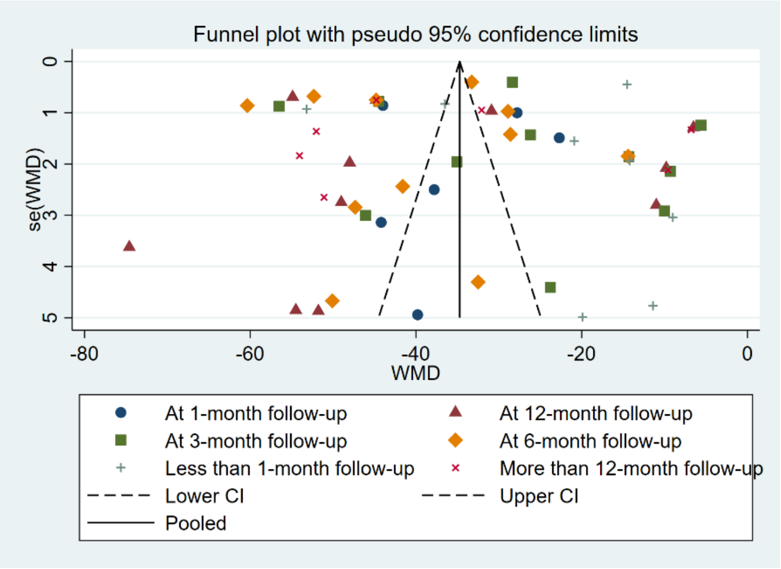

FIGURE 5 ODI grouping funnel plot
