# Supplementary material for: Safety and clinical efficacy of endoscopic procedures for the treatment of adjacent segmental disease after lumbar fusion: A systematic review and meta-analysis
Source: PLoS One. 2023 Feb 6;18(2):e0280135. doi: 10.1371/journal.pone.0280135 (PMC9901788; doi:10.1371/journal.pone.0280135)
Supplement: S2 Fig — (PDF) [file pone.0280135.s003.pdf]

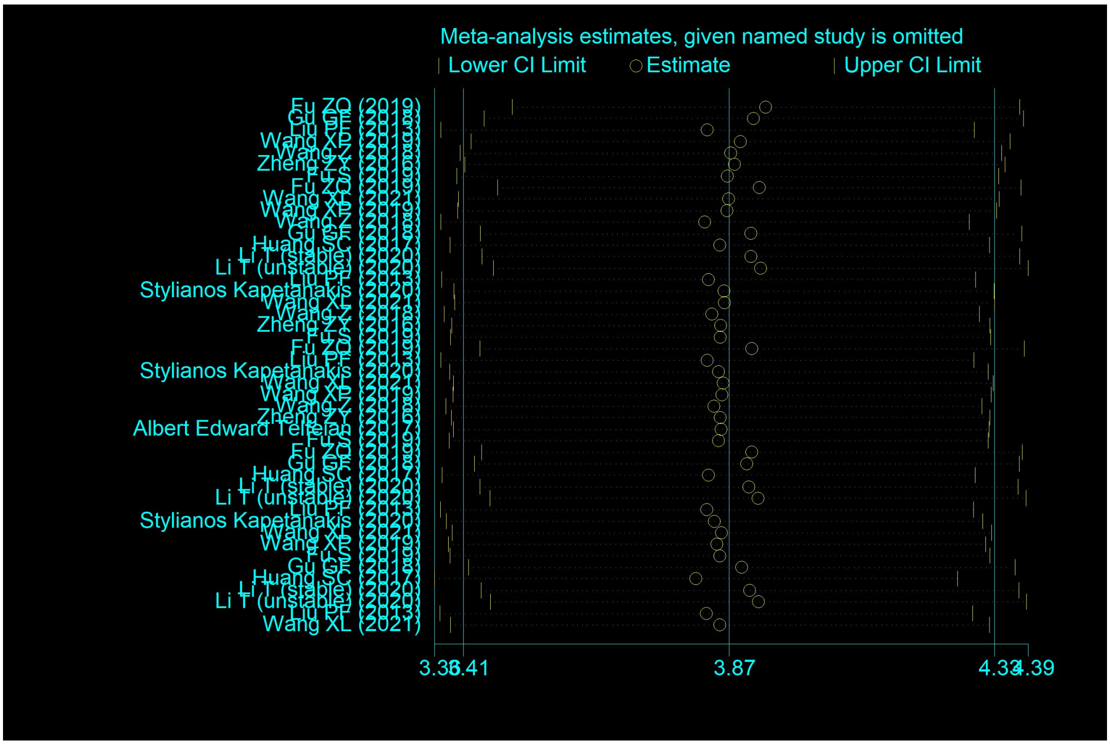

Fig S2-1. Sensitivity analysis graph of VAS-back

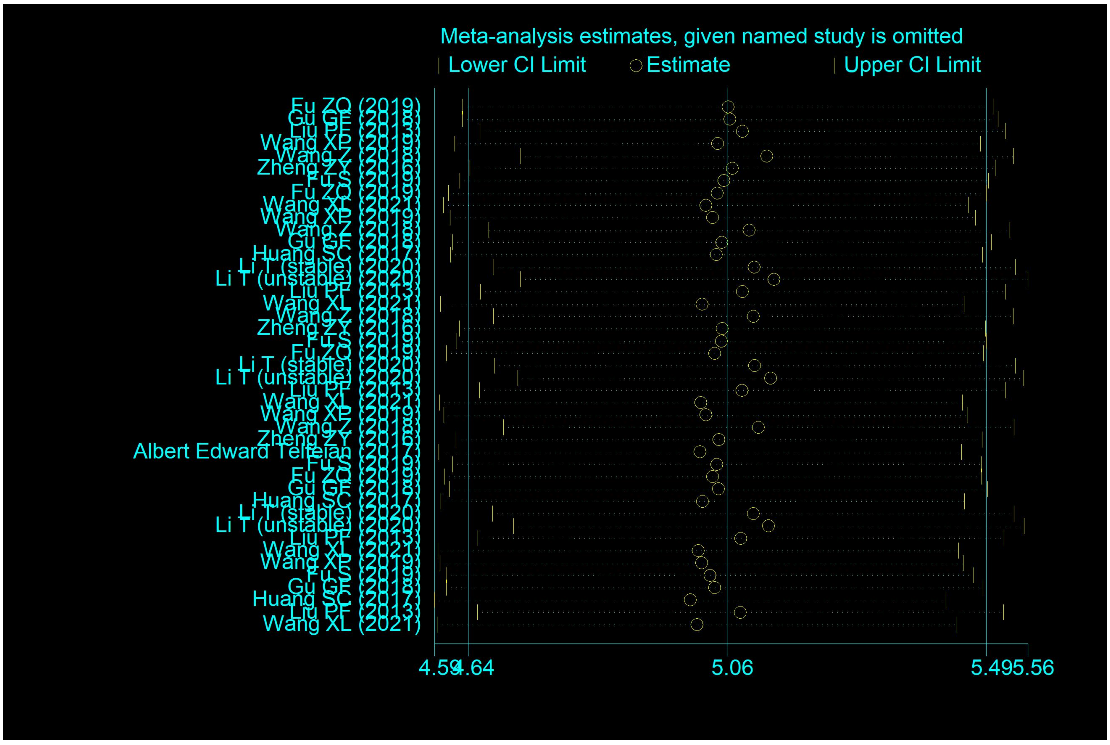

Fig S2-2. Sensitivity analysis graph of VAS-leg

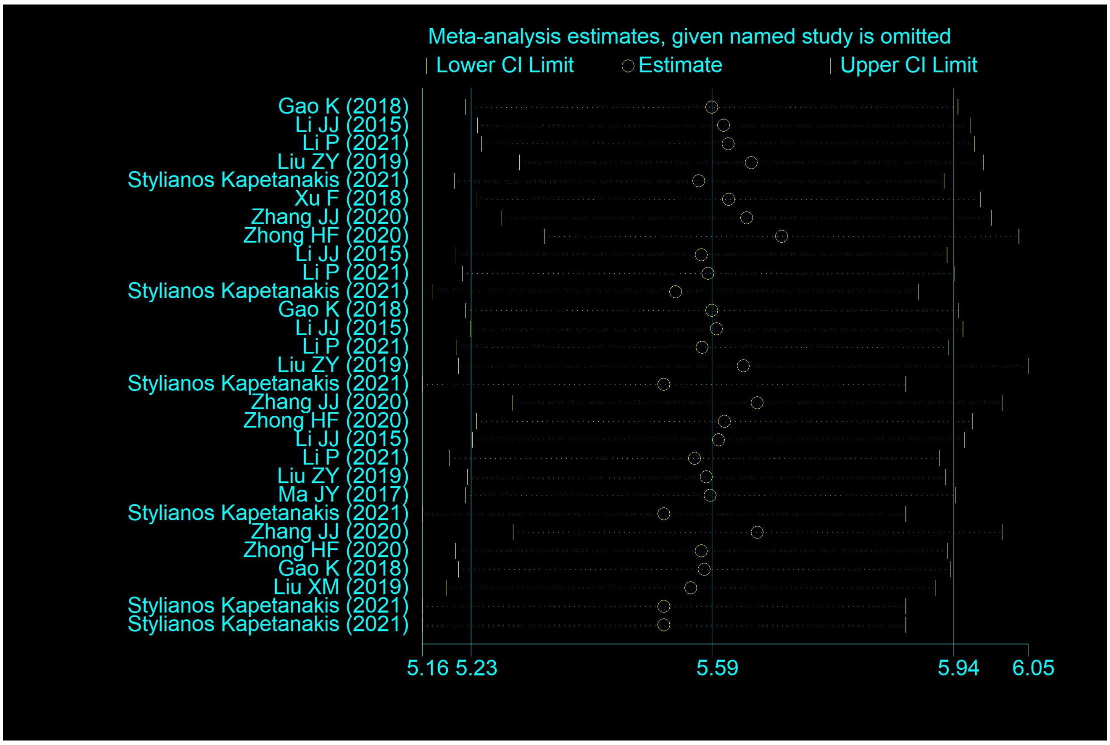

Fig S2-3. Sensitivity analysis graph of VAS-mix

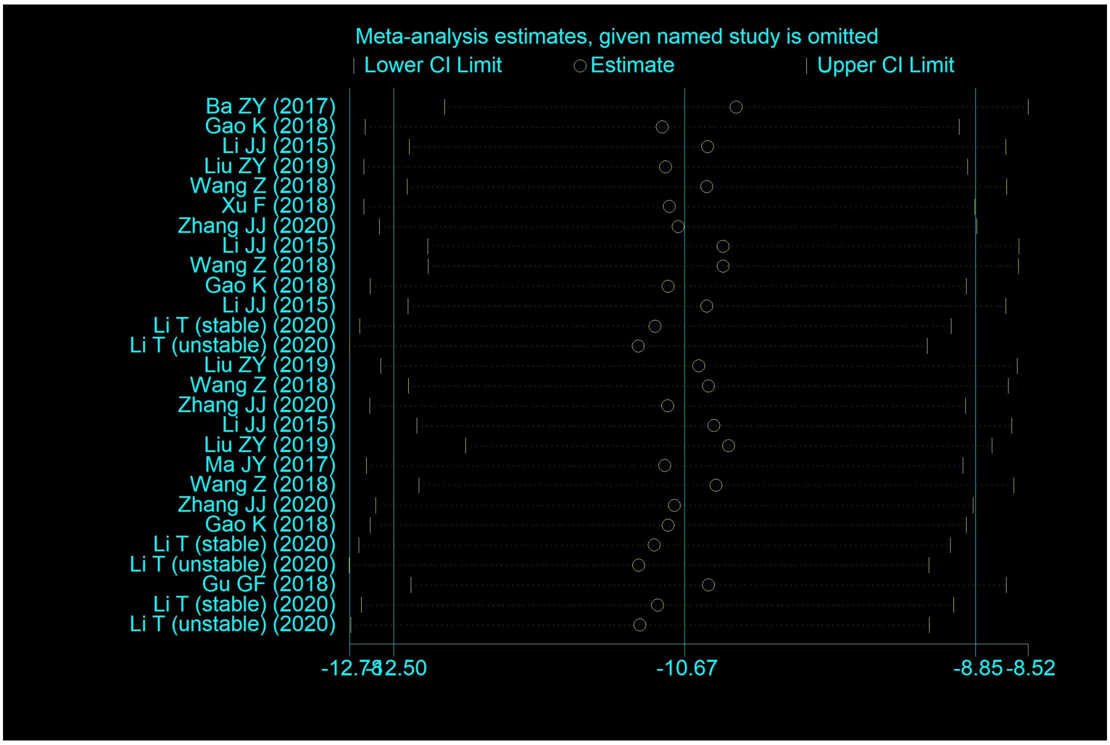

Fig S2-4. Sensitivity analysis graph of JOA

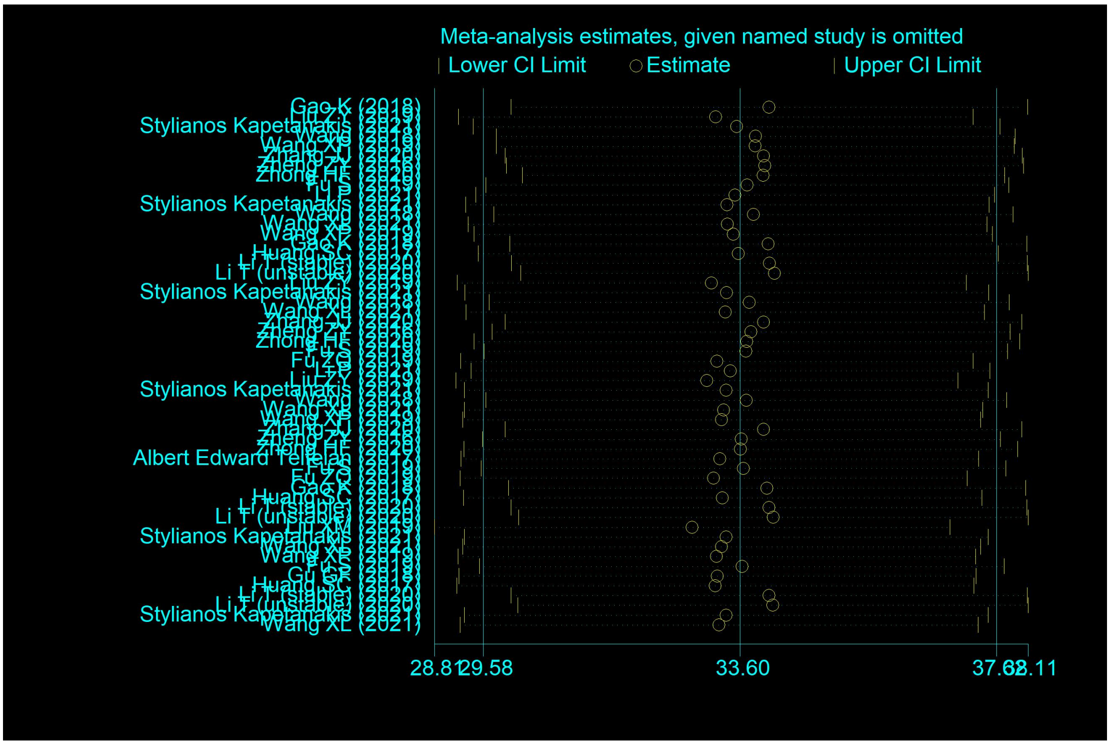

Fig S2-5. Sensitivity analysis graph of ODI
