# Supplementary material for: Safety and clinical efficacy of endoscopic procedures for the treatment of adjacent segmental disease after lumbar fusion: A systematic review and meta-analysis
Source: PLoS One. 2023 Feb 6;18(2):e0280135. doi: 10.1371/journal.pone.0280135 (PMC9901788; doi:10.1371/journal.pone.0280135)
Supplement: S3 Fig — (PDF) [file pone.0280135.s004.pdf]

Study or Subgroup	Control Mean	Control SD	Control Total	Experimental Mean	Experimental SD	Experimental Total	Weight	Std. Mean Difference IV, Random, 95% CI
Albert Edward Telfeian 2017	8.22	1.2	9	3.5	2.93	9	8.0%	2.01 [0.82, 3.19]
Fu S 2019	6.99	1.54	47	2.43	0.81	47	9.4%	3.68 [3.00, 4.35]
Fu ZQ 2019	3.44	0.6	31	1.28	1.04	31	9.3%	2.51 [1.84, 3.19]
Gu GF 2018	4.12	0.97	25	1.21	0.31	25	8.6%	3.98 [2.99, 4.96]
Huang SC 2017	7.29	1.36	28	0.89	0.74	28	7.9%	5.76 [4.54, 6.99]
Li T 2020	4.7	1.6	20	2.5	1.1	20	9.3%	1.57 [0.85, 2.29]
Liu PF 2013	6.57	1.34	20	0.94	0.61	20	7.5%	5.30 [3.93, 6.67]
Stylianos Kapetanakis 2020	8.53	0.83	15	3.53	0.83	15	6.5%	5.86 [4.12, 7.61]
Wang XL 2021	6.15	1.23	19	1.58	0.5	19	7.7%	4.77 [3.47, 6.06]
Wang XP 2019	6.62	1.33	18	1.79	1.17	18	8.2%	3.77 [2.64, 4.90]
Wang Z 2018	7.86	1.42	50	2.83	1.07	50	9.3%	3.97 [3.29, 4.65]
Zheng ZY 2016	6.77	1.09	22	1.81	1.17	22	8.2%	4.31 [3.19, 5.42]
**Total (95% CI)**			**304**			**304**	**100.0%**	**3.86 [3.13, 4.60]**

Heterogeneity: $Tau^2 = 1.41$; $Chi^2 = 77.68$, df = 11 (P < 0.00001); $I^2 = 86\%$
Test for overall effect: Z = 10.24 (P < 0.00001)

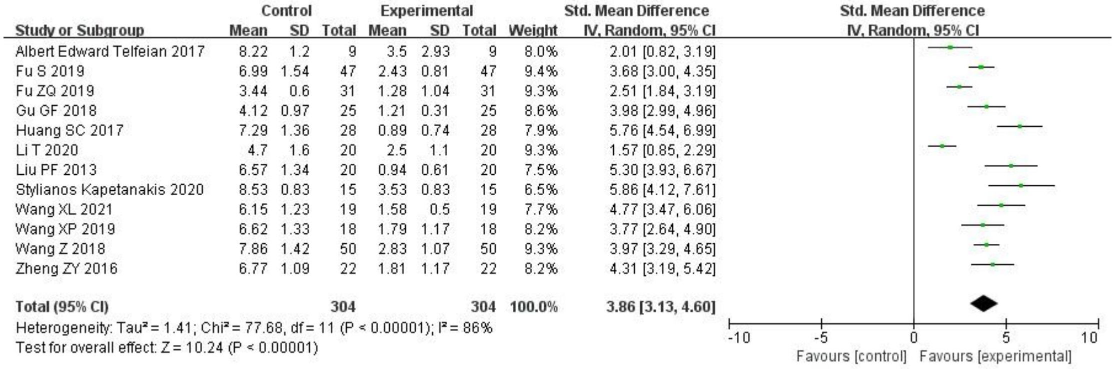

Fig S3-1. Heterogeneity test for VAS-back forest plots

Study or Subgroup	Control Mean	Control SD	Control Total	Experimental Mean	Experimental SD	Experimental Total	Weight	Std. Mean Difference IV, Random, 95% CI
Albert Edward Telfeian 2017	8.44	1.24	9	1.25	1.39	9	8.2%	5.20 [3.07, 7.33]
Fu S 2019	8.43	1.88	47	2.21	0.44	47	9.9%	4.52 [3.74, 5.29]
Fu ZQ 2019	7.83	0.71	31	1.83	0.38	31	8.5%	10.40 [8.45, 12.36]
Gu GF 2018	7.81	0.79	25	1.92	0.22	25	8.2%	10.00 [7.88, 12.11]
Huang SC 2017	8.39	1.07	28	0.86	0.71	28	8.9%	8.18 [6.52, 9.83]
Li T 2020	6.2	1.5	20	3	1.2	20	9.8%	2.31 [1.49, 3.13]
Liu PF 2013	5.12	1.25	20	0.96	0.64	20	9.5%	4.11 [2.97, 5.24]
Wang XL 2021	8.56	1.07	19	1.46	0.47	19	8.2%	8.41 [6.32, 10.51]
Wang XP 2019	8.42	1.71	18	1.53	0.67	18	9.2%	5.19 [3.76, 6.62]
Wang Z 2018	5.62	1.06	50	2.65	1.28	50	10.0%	2.51 [1.98, 3.04]
Zheng ZY 2016	7.63	2.04	22	1.62	0.4	22	9.6%	4.02 [2.96, 5.07]
**Total (95% CI)**			**289**			**289**	**100.0%**	**5.72 [4.34, 7.11]**

Heterogeneity: $Tau^2 = 4.92$; $Chi^2 = 166.07$, df = 10 (P < 0.00001); $I^2 = 94\%$
Test for overall effect: Z = 8.09 (P < 0.00001)

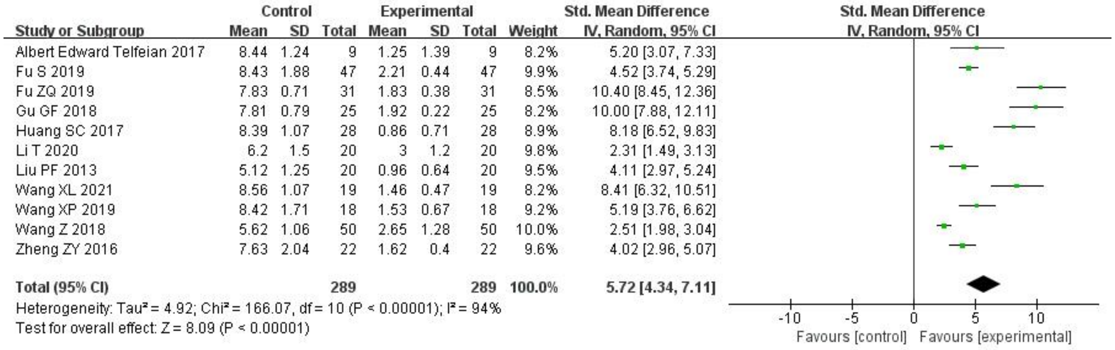

Fig S3-2. Heterogeneity test for VAS-leg forest plots

Study or Subgroup	Control Mean	Control SD	Control Total	Experimental Mean	Experimental SD	Experimental Total	Weight	Std. Mean Difference IV, Random, 95% CI
Gao K 2018	7.5	1.2	20	1.6	0.5	20	11.1%	6.29 [4.71, 7.87]
Li JJ 2015	8.13	1.21	31	2.81	1.49	31	11.5%	3.87 [3.01, 4.73]
Li P 2021	7.82	1.24	26	1.54	0.51	26	11.2%	6.52 [5.11, 7.94]
Liu XM 2019	8.4	2.8	33	1.8	0.6	33	11.5%	3.22 [2.48, 3.97]
Liu ZY 2019	7.13	0.56	201	1.38	0.22	201	11.5%	13.49 [12.53, 14.45]
Ma JY 2017	7.83	2.12	24	2.16	1.43	24	11.5%	3.08 [2.23, 3.94]
Stylianos Kapetanakis 2021	8.86	0.69	7	1.14	0.9	7	8.9%	9.01 [4.94, 13.09]
Zhang JJ 2020	6.7	1.2	15	3.2	1.3	15	11.4%	2.72 [1.69, 3.75]
Zhong HF 2020	7.25	1.25	36	1.25	0.41	36	11.4%	6.38 [5.21, 7.55]
**Total (95% CI)**			**393**			**393**	**100.0%**	**5.99 [3.50, 8.48]**

Heterogeneity: $Tau^2 = 13.85$; $Chi^2 = 382.38$, df = 8 (P < 0.00001); $I^2 = 98\%$
Test for overall effect: Z = 4.71 (P < 0.00001)

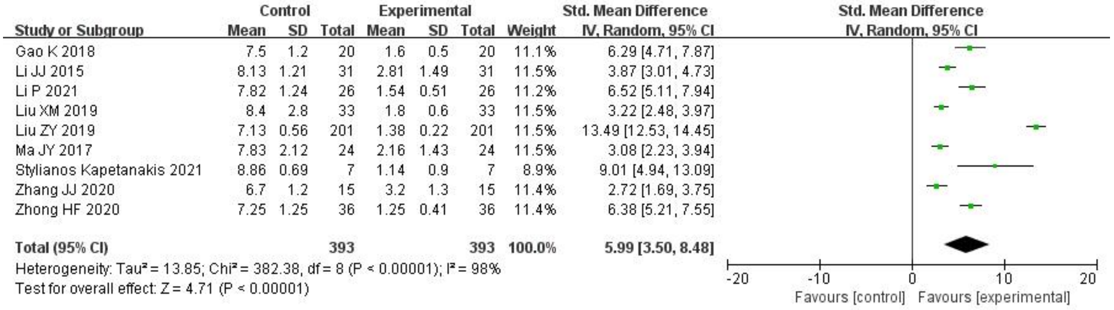

Fig S3-3. Heterogeneity test for VAS-mix forest plots

Study or Subgroup	Control Mean	Control SD	Control Total	Experimental Mean	Experimental SD	Experimental Total	Weight	Std. Mean Difference IV, Random, 95% CI
Ba ZY 2017	5.8	1.32	33	25	5.42	33	8.3%	-4.81 [-5.78, -3.84]
Gao K 2018	20	4	20	28	2.1	20	8.4%	-2.45 [-3.29, -1.61]
Gu GF 2018	9.71	2.24	25	24.41	5.71	25	8.4%	-3.34 [-4.21, -2.46]
Hiroki Iwai 2019	10.5	4.2	13	16.1	4.9	13	8.4%	-1.19 [-2.03, -0.34]
Li JJ 2015	10.77	4.21	31	26.3	3.55	31	8.4%	-3.94 [-4.81, -3.07]
Li T 2020	15.9	4.6	20	22	3.6	20	8.5%	-1.45 [-2.15, -0.74]
Liu XM 2019	24.6	8.4	33	5.4	2.3	33	8.5%	3.08 [2.36, 3.81]
Liu ZY 2019	10.71	4.18	201	28.45	2.31	201	8.6%	-5.24 [-5.66, -4.83]
Ma JY 2017	9.82	2.76	42	17.28	4.11	42	8.5%	-2.11 [-2.65, -1.57]
Wang Z 2018	10.63	4.17	50	26.43	3.62	50	8.5%	-4.02 [-4.71, -3.33]
Xu F 2018	9.95	0.62	31	28.2	2.08	31	7.3%	-11.74 [-13.94, -9.55]
Zhang JJ 2020	14.7	2.9	15	23.7	2.5	15	8.2%	-3.23 [-4.37, -2.10]
**Total (95% CI)**			**514**			**514**	**100.0%**	**-3.27 [-4.76, -1.78]**

Heterogeneity: $Tau^2 = 6.67$; $Chi^2 = 519.09$, df = 11 (P < 0.00001); $I^2 = 98\%$
Test for overall effect: Z = 4.31 (P < 0.0001)

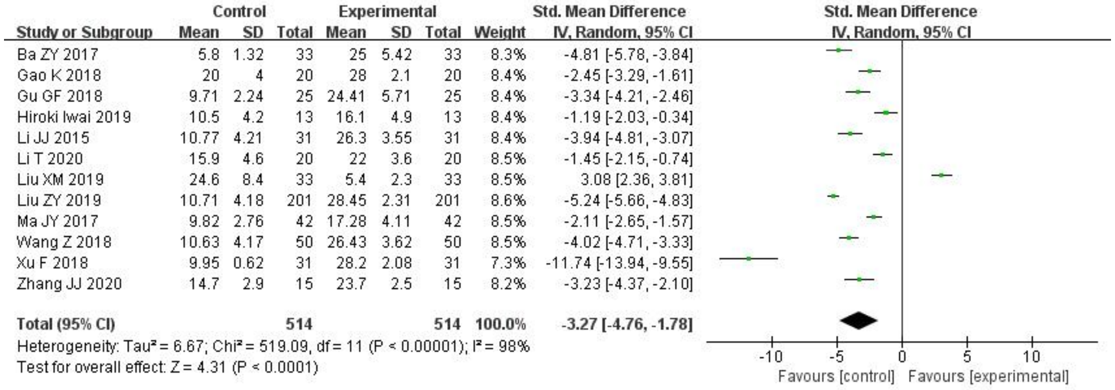

Fig S3-4. Heterogeneity test for JOA forest plots

Study or Subgroup	Control Mean	Control SD	Control Total	Experimental Mean	Experimental SD	Experimental Total	Weight	Std. Mean Difference IV, Random, 95% CI
Zhong HF 2020	39.72	2.37	36	6.45	0.39	36	5.5%	19.38 [16.09, 22.67]
Zheng ZY 2016	70.8	16.3	22	23.7	13.4	22	6.9%	3.10 [2.20, 4.00]
Zhang JJ 2020	30	7.1	15	15.6	0.9	15	6.9%	2.77 [1.73, 3.81]
Wang XP 2019	73.2	16.2	18	18.7	12.7	18	6.8%	3.66 [2.55, 4.77]
Wang XL 2021	74.44	10.63	19	23.33	4.49	19	6.6%	6.13 [4.54, 7.72]
Stylianos Kapetanakis 2021	60.9	1.5	7	16.1	1.3	7	1.3%	29.88 [16.78, 42.98]
Liu ZY 2019	75.54	11.84	201	15.18	2.87	201	7.0%	6.99 [6.47, 7.52]
Liu XM 2019	89.2	17	33	14.6	12	33	6.9%	5.01 [4.01, 6.02]
Li T 2020	23.2	7.7	20	13.6	5.5	20	7.0%	1.41 [0.71, 2.11]
Li P 2021	75.4	9.6	28	33.8	8.6	28	6.9%	4.50 [3.49, 5.51]
Huang SC 2017	70.93	8.72	28	16.86	4.27	28	6.6%	7.77 [6.18, 9.35]
Gu GF 2018	65.32	6.07	25	13.27	3.07	25	6.2%	10.65 [8.41, 12.90]
Gao K 2018	18	11	20	7	6	20	7.0%	1.22 [0.54, 1.90]
Fu ZQ 2019	69.22	3.37	31	14.33	1.85	31	5.2%	19.94 [16.28, 23.60]
Fu S 2019	40.78	6.32	47	8.69	1.45	47	6.8%	6.94 [5.85, 8.03]
Albert Edward Telfeian 2017	65.78	11.55	9	14	8.94	9	6.4%	4.77 [2.78, 6.77]
**Total (95% CI)**			**559**			**559**	**100.0%**	**6.82 [5.20, 8.44]**

Heterogeneity: Tau² = 9.68; Chi² = 504.83, df = 15 (P < 0.00001); I² = 97%

Test for overall effect: Z = 8.25 (P < 0.00001)

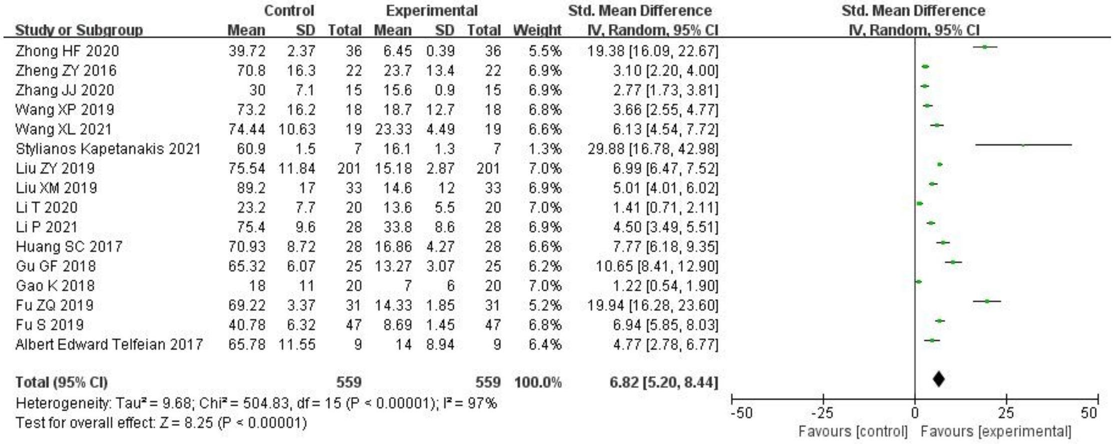

Fig S3-5. Heterogeneity test for ODI forest plots
