# Supplementary material for: Safety and clinical efficacy of endoscopic procedures for the treatment of adjacent segmental disease after lumbar fusion: A systematic review and meta-analysis
Source: PLoS One. 2023 Feb 6;18(2):e0280135. doi: 10.1371/journal.pone.0280135 (PMC9901788; doi:10.1371/journal.pone.0280135)
Supplement: S1 File — S1 Table. Observation time nodes of different observation indexes in the 24 studies. S2 Table. Meta-regression analysis of observation time nodes in this study. (DOC) [file pone.0280135.s005.doc]

# Safety and clinical efficacy of endoscopic procedures for the treatment of adjacent segmental disease after lumbar fusion: a systematic review and meta-analysis

**Nan Wang^1[^, Xiyu Liu^1[^, Yimin Xie^1[^, Yawei Zheng^2^, Zhipeng Xi^1^, Wenqiang Xu^1^, Rongrong Deng^1^, Tian Tang^1*^ and Xin Liu^1*^**

^1^Department of Spine Surgery, Affiliated Hospital of Integrated Traditional Chinese and Western Medicine for Nanjing University of Chinese Medicine, Nanjing, Jiangsu Province 210028 P.R. China.

^2^Department of Cardiovascular Medicine, Jiangsu Provincial Hospital of Traditional Chinese Medicine, Nanjing, Jiangsu Province 210000 P.R. China.

[These authors contributed equally to this work.

*tangtian881022@126.com(TT); [liuxin@njucm.edu.cn(XL)](mailto:liuxin@njucm.edu.cn(XL))

**Table S1** **Observation time nodes of different observation indexes in the 24 studies.**

| **NO** | **Trial** | **NRS**  **(month)** | **mJOA**  **(month)** | **VAS**  **(month)** | **VAS(back,leg)(month)** | **ODI**  **(month)** | **JOA**  **(month)** | **MacMab**  **(month)** |
| --- | --- | --- | --- | --- | --- | --- | --- | --- |
| 1 | Hiroki Iwai 2019(20) | 13.1 | 13.1 | \ | \ | \ | \ | \ |
| 2 | Xiaoming Liu 2019(21) | \ | 12 | 12 | \ | 12 | \ | \ |
| 3 | Guangfei Gu 2018(22) | \ | \ | \ | <1,3,12,>12 | >12 | >12 | >12 |
| 4 | Albert Edward Telfeian 2017(23) | \ | \ | \ | 12 | 12 | \ | \ |
| 5 | Wang Xuepeng 2019(24) | \ | \ | \ | <1,1,6,12 | <1,1,6,12 | \ | 12 |
| 6 | Liu Zuwang 2019(25) | \ | \ | <1,3,6 | \ | <1,3,6 | <1,3,6 | 12 |
| 7 | Ma Jiye 2017(26) | \ | \ | 6 | \ | \ | 6 | \ |
| 8 | Li Jianjiang 2015(27) | \ | \ | <1,1,3,6 | \ | \ | <1,1,3,6 | \ |
| 9 | Zheng Zhenyang 2016(28) | \ | \ | \ | <1,3,6,9 | <1,3,6,9 | \ | 9 |
| 10 | Wang Xiaoli 2021(29) | \ | \ | \ | 1,3,6,12,>12 | 1,3,6,12,>12 | \ | >12 |
| 11 | Xu Feng 2018(30) | \ | \ | <1,>12 | \ | \ | <1,>12 | >12 |
| 12 | Fu Song 2019(31) | \ | \ | \ | 1,6,12,>12 | 1,6,12,>12 | \ | >12 |
| 13 | Wang Zhen 2018(32) | \ | \ | \ | 1,6,12,>12 | 1,6,12,>12 | 1,6,12,>12 | \ |
| 14 | Huang Shenchang 2017(33) | \ | \ | \ | 3,12,>12 | 3,12,>12 | \ | >12 |
| 15 | Zhang Jianjun 2020(34) | \ | \ | <1,3,>12 | \ | <1,3,>12 | <1,3,>12 | >12 |
| 16 | Liu Pengfei 2013(35) | \ | \ | \ | <1,3,12,>12 | \ | \ | >12 |
| 17 | Gao Kun 2018(36) | \ | \ | <1,3,12 | \ | <1,3,12 | <1,3,12 | \ |
| 18 | Zhong Honghua 2020(37) | \ | \ | <1,3,6 | \ | <1,3,6 | \ | \ |
| 19 | Zhaoyu Ba 2017(15) | \ | \ | \ | \ | <1 | <1 | \ |
| 20 | Tong Li 2020(38) | \ | \ | \ | 3,12,>12 | 3,12,>12 | 3,12,>12 | \ |
| 21 | Fu Zhongquan  2019(39) | \ | \ | \ | <1,1,6,12 | 6,12 | \ | \ |
| 22 | Li Peng 2021(40) | \ | \ | <1,1,3,6 | \ | 1,6 | \ | \ |
| 23 | Stylianos Kapetanakis 2020(41) | \ | \ | \ | 1,5,3,6,12 | \ | \ | \ |
| 24 | Stylianos Kapetanakis 2021(42) | \ | \ | <1,1,3,6,12,>12 | <1,1,3,6,12,>12 | \ | \ | \ |

**Table S2 Meta-regression analysis of observation time nodes in this study.**

| **Postoperative Outcomes** | **Coef.** | **Std. Err.** | **t** | **P>|t|** | **95% Conf. Interval** | |
| --- | --- | --- | --- | --- | --- | --- |
| VAS-back | .0172673 | .0270318 | 0.64 | 0.526 | -.0372117 | .0717462 |
| VAS-leg | .0667005 | .0312443 | 2.13 | 0.039 | .0036013 | .1297996 |
| VAS-mix | .1229076 | .0406609 | 3.02 | 0.005 | .0394782 | .2063369 |
| _JOA | .2112702 | .1256017 | 1.68 | 0.105 | -.0474114 | .4699518 |
| ODI | .4255352 | .3114844 | 1.37 | 0.178 | -.199504 | 1.050574 |

Note: Coef: Coefficient; Std. Err: Standard error; Conf. Interval: Confidence interval.
